# Use of the Chatbot “Vivibot” to Deliver Positive Psychology Skills and Promote Well-Being Among Young People After Cancer Treatment: Randomized Controlled Feasibility Trial

**DOI:** 10.2196/15018

**Published:** 2019-10-31

**Authors:** Stephanie Greer, Danielle Ramo, Yin-Juei Chang, Michael Fu, Judith Moskowitz, Jana Haritatos

**Affiliations:** 1 Hopelab San Francisco, CA United States; 2 Department of Psychiatry, Weill Institute for Neurosciences University of California, San Francisco San Francisco, CA United States; 3 Feinberg School of Medicine Northwestern University Chicago, IL United States

**Keywords:** chatbot, positive psychology, young adult, cancer

## Abstract

**Background:**

Positive psychology interventions show promise for reducing psychosocial distress associated with health adversity and have the potential to be widely disseminated to young adults through technology.

**Objective:**

This pilot randomized controlled trial examined the feasibility of delivering positive psychology skills via the *Vivibot* chatbot and its effects on key psychosocial well-being outcomes in young adults treated for cancer.

**Methods:**

Young adults (age 18-29 years) were recruited within 5 years of completing active cancer treatment by using the *Vivibot* chatbot on Facebook messenger. Participants were randomized to either immediate access to *Vivibot* content (experimental group) or access to only daily emotion ratings and access to full chatbot content after 4 weeks (control). Created using a human-centered design process with young adults treated for cancer, *Vivibot* content includes 4 weeks of positive psychology skills, daily emotion ratings, video, and other material produced by survivors, and periodic feedback check-ins. All participants were assessed for psychosocial well-being via online surveys at baseline and weeks 2, 4, and 8. Analyses examined chatbot engagement and open-ended feedback on likability and perceived helpfulness and compared experimental and control groups with regard to anxiety and depression symptoms and positive and negative emotion changes between baseline and 4 weeks. To verify the main effects, follow-up analyses compared changes in the main outcomes between 4 and 8 weeks in the control group once participants had access to all chatbot content.

**Results:**

Data from 45 young adults (36 women; mean age: 25 [SD 2.9]; experimental group: n=25; control group: n=20) were analyzed. Participants in the experimental group spent an average of 74 minutes across an average of 12 active sessions chatting with *Vivibot* and rated their experience as helpful (mean 2.0/3, SD 0.72) and would recommend it to a friend (mean 6.9/10; SD 2.6). Open-ended feedback noted its nonjudgmental nature as a particular benefit of the chatbot. After 4 weeks, participants in the experimental group reported an average reduction in anxiety of 2.58 standardized t-score units, while the control group reported an increase in anxiety of 0.7 units. A mixed-effects models revealed a trend-level (*P*=.09) interaction between group and time, with an effect size of 0.41. Those in the experimental group also experienced greater reductions in anxiety when they engaged in more sessions (z=–1.9, *P*=.06). There were no significant (or trend level) effects by group on changes in depression, positive emotion, or negative emotion.

**Conclusions:**

The chatbot format provides a useful and acceptable way of delivering positive psychology skills to young adults who have undergone cancer treatment and supports anxiety reduction. Further analysis with a larger sample size is required to confirm this pattern.

## Introduction

A total of 70,000 adolescents and young adults, aged 15-39 years, are diagnosed with cancer each year, making cancer the leading cause of disease-related death in young people in the United States [1]. In addition to this disproportionate disease burden, there are significant and unique psychosocial needs for adolescents and young adults diagnosed with cancer [2]. Adolescents and young adults are most likely to experience depression, heightened anxiety, distress, and posttraumatic stress disorder in their first 12-24 months after completing treatment, which poses significant unmet mental health needs in the period after cancer treatment [2-4]. 

Several studies have suggested that 20%-30% of young people treated for cancer report moderate-to-severe psychological distress lasting into adulthood [3]. Compared to their siblings, adolescents and young adults with cancer have reported poorer overall mental health [5] and are twice as likely to report clinical levels of emotional distress [6-8]. Despite this disproportionate need, there remains a lack of age-appropriate psychosocial support for adolescents and young adults after cancer treatment, which is further complicated by the difficulty reaching this geographically dispersed population [9]. There is therefore a need to address the unmet mental health burden experienced by young people who have been diagnosed with cancer in the initial years after treatment.

Advances in the field of positive psychology have shown promise in addressing the psychological distress associated with health adversity [10]. This work is built upon the importance of positive emotion in the maintenance of psychological and physical well-being [11] and evidence that positive emotion is uniquely associated with a lower risk of morbidity and mortality in healthy and chronically ill samples, independent of the effects of negative emotion [12-15]. Interventions that explicitly target positive emotion show promise for improving health outcomes in a number of chronic illnesses, including diabetes [16,17], heart disease [18,19], hypertension [20,21], depression [22,23], and HIV [24].

In the area of adolescent and young adult cancer survivorship, there is evidence that several personal resources have been shown to mitigate negative and promote positive psychosocial outcomes among young people treated for cancer [25-27]. For example, the *Promoting Resilience in Stress Management* (PRISM) intervention was designed to promote resilience coping skills in adolescents and young adults with a cancer diagnosis. A pilot trial demonstrated the feasibility of an in-person PRISM intervention with this population [28], and a clinical trial testing the efficacy of the PRISM skills intervention compared to usual care among adolescents and young adults after cancer treatment, aged 12-25 years (N=100), showed statistically significant improvements in patient‐reported resilience and cancer‐specific quality of life, as well as reduced psychological distress [29]. Secondary analyses showed additional value of benefit finding and hope among those who got PRISM with moderate-to-large effect sizes [30]. Interventions targeting resilience and positive emotion have the potential to support mental health needs among young people treated for cancer.

Depression is common among young people with cancer [31], and interventions based on increasing positive emotion have been shown to relieve symptoms of depression in a meta-analysis [23]. Psychological interventions that specifically target depression in people with cancer have shown some efficacy but tend to be underutilized by younger adults [31]. Anxiety is also common among young people with cancer, with effective treatments either not available in the settings in which adolescents and young adults with cancer receive treatment or not offered by referring clinicians. [31]. In the absence of treatments that support adolescents and young adults after cancer treatment, there is a need to fill the gap.

Digital health interventions may extend the reach and impact of positive psychology skills interventions among young people treated for cancer. Within the vast array of digital intervention platforms, fully automated conversational agents (“chatbots”) have the distinct advantage of being perceived as accessible to youth [32] and can deliver a structured set of content that can simulate the content experienced by real-life conversation (eg, with a supportive friend).

The literature on the use of chatbots to stimulate conversation about health or to actually change health behavior is emerging. For example, Bickmore et al [33] demonstrated that a carefully designed health-related conversational agent could establish a therapeutic relationship with adults attempting to increase exercise. A review of 14 chatbots in health care found that their use is still rare relative to other areas [34]. Further, most studies designed to evaluate these chatbots were quasi-experimental in design and lack outcome measures that were clear health indicators. The notable exception was the trial evaluating the *Woebot* chatbot, which found a significant reduction in depression symptoms after 2 weeks of use in college students seeking mental health support [35]. More research is needed to understand whether chatbots are associated with changes in health behaviors and emotional distress in specific populations and through varied media including social media.

Building on evidence-based interventions in the field of positive psychology and using a human-centered design approach, Hopelab (San Francisco, CA) created a chatbot called *Vivibot*, delivered over Facebook messenger, to address psychosocial needs of young people treated for cancer. The purpose of this feasibility study was to evaluate engagement and usability of the *Vivibot* chatbot. An additional goal was to evaluate the preliminary effects of positive psychology skills delivered through *Vivibot* on key psychosocial well-being outcomes in young adults treated for cancer. Outcomes were compared across two conditions: 4 weeks of *Vivibot* exposure (experimental group) or access to daily emotion ratings through Facebook Messenger with access to the full *Vivibot* chatbot after 4 weeks (control group).

Our hypotheses were as follows: (1) After 4 weeks, exposure to *Vivibot* would result in decreased depression, anxiety, and negative emotions and increased positive emotions compared to the control condition. (2) Among the treatment group, greater engagement in chatbot lessons would be associated with better outcomes.

## Methods

### Study Design

This was a 4-week pilot randomized controlled trial evaluating feasibility, usability, and initial efficacy of the *Vivibot* chatbot. At 4 weeks, control participants were given full access to the content in the experimental condition. An 8-week follow-up survey thus allowed for validation of the main outcome analyses in the control group.

### Participants and Recruitment

Participants were young adults, aged 18-29 years, consistent with the developmental literature [36] and the field of oncology [4]; English literate; and reported having a cancer diagnosis and completing treatment for cancer within 5 years of starting the study. They also had to have access to Facebook Messenger for the study duration (either through a Facebook or Instagram account). Participants were excluded if they did not meet age or cancer diagnosis or treatment requirements or were unable to access *Vivibot* through Facebook Messenger. They were not excluded based on the type of cancer diagnosis.

Recruitment was conducted through Facebook advertising (67% of final sample), survivorship organizations (15%), and direct email after a potential participant expressed interest at a conference or event (17%). Enrollment was managed entirely through the *Vivibot* chatbot. During the recruitment period, once a user opened the chatbot interface for the first time, he/she was automatically asked four screening questions to determine eligibility. Those eligible and interested were sent an identification code and link to the study consent survey.

### Study Procedure

The Ethical and Independent Review Services [37] board approved all study procedures. Following completion of the baseline assessment, participants were instructed to return to *Vivibot* and indicate that they completed the baseline survey. The chatbot then randomized users 1:1 to one of two groups: (1) immediate access to the full *Vivibot* chatbot content (experimental group) or (2) access to daily emotion ratings through Facebook Messenger with delayed full access to the full *Vivibot* chatbot only after 4 weeks (control group).

Online assessments through Qualtrics software (Salt Lake City, UT) were administered at baseline and weeks 2, 4, and 8. Participants received US $20 Amazon gift cards for completing each survey (a total of US $80 possible compensation). After 4 weeks, the control group participants were given access to all of the *Vivibot* content. After 8 weeks, participation in the study was complete, but all participants were informed that they could continue to use the chatbot product as much or as little as they desired.

### Intervention Conditions

#### Vivibot

*Vivibot* is a chatbot designed to deliver prewritten and automatically delivered material to users online via a decision tree structure. Before study enrollment, users were explicitly told that they are chatting with an automated system (not a person) and periodically reminded of this throughout study participation.

*Vivibot* delivers a cognitive and behavioral intervention to increase positive emotion developed by Moskowitz et al (eg, [24]). The intervention was originally based on the Stress and Coping theory and the Broaden-and-Build theory of positive emotion and focused on the teaching and practice of eight positive psychological skills: noticing and acknowledging positive events, savoring positive events, gratitude, positive reappraisal, acts of kindness, mindfulness, personal strengths, and attainable goals. Rationale for inclusion of each of the positive emotion skills is provided elsewhere [38]. This core intervention was adapted for the chatbot format by creating seven conversational teaching lessons and seven practice lessons that were repeated three times to create 28 days of content. The eight skills were covered in seven lessons by combining acknowledging and savoring positive events into one lesson set.

Before launching the pilot trial, Hopelab conducted formative work through interviews and focus groups with adolescents and young adults treated for cancer to refine content for the chatbot format and inform adaptation for delivery to a young userbase with a shared experience of cancer treatment. Upon completion of the focus groups, text was tailored based on their specific suggestions, and video and other content produced by young adults who were treated for cancer was incorporated directly into the chatbot. *Vivibot* additionally incorporated six daily emotion ratings (described in the *Measures* section) and periodic check-ins on participants’ satisfaction with their interactions with the chatbot. The content is outlined in Multimedia Appendix 1, and sample user experience content is in Multimedia Appendix 2.

#### Control

Control participants received delayed access to the full *Vivibot* chatbot. Those randomized to this group were given a message within the chatbot saying that their access to the full content would be delayed by 4 weeks. During this time, they were asked to report daily emotion ratings but received no other chatbot content. A set of six participants in the control condition encountered a technical error after the 2-week survey. This resulted in access to the full chatbot content at 2 weeks instead of the full 4 weeks. These six participants have been excluded from reported analyses to test outcomes at 4 weeks across a clean sample.

### Measures

#### Chatbot Feasibility/Acceptability

##### Engagement With the Chatbot

Full conversational history with the chatbot was examined by session. An interaction was considered a *session* if there was engagement with the bot lasting at least two user inputs within 5 minutes and a break no longer than 5 minutes. The *total number of sessions* was calculated as the count of all sessions for an individual user, and the *total interaction time* with the chatbot was defined as the total time from the start to the end of a session summed across all sessions. In addition, an *engaged session* was defined as any session that included, at minimum, a completion of the 6-item emotion rating. Identifying engaged sessions was important because some interactions deemed *sessions* included only a participant receiving a notification to check-in and then responding that they were not available to talk. These sessions therefore did not include meaningful interactions with the chatbot content. Defining engaged sessions as requiring a user to progress to daily emotion rating completion allowed for comparisons of engaged sessions in both experimental and control groups.

##### Chatbot Feedback

At the completion of each skill in the experimental group, users were asked to rate how helpful they found the lesson of the day on a scale from 0 (“not really”) to 3 (“yes, very”). Mean ratings across all lessons were calculated for each participant (a given participant could have more than one rating). Participants in the experimental group were also given periodic opportunities to provide open-ended feedback about the chatbot program. On the seventh interaction, participants were specifically asked “How likely would you be to recommend *Vivibot* to a friend?” (rated on a scale from 0 to 10) and “Why did you give that score?” These questions were used to assess how much participants enjoyed the chatbot and what they found particularly valuable or not valuable.

#### Well-Being Outcomes

##### Anxiety and Depression Symptoms

Anxiety and depression symptoms were measured by assessments from the Patient-Reported Outcomes Measurement Information System (PROMIS) initiative [39]. PROMIS measures are listed as “emerging measures” for further research by the American Psychiatric Association and published in the Diagnostic and Statistical Manual of Mental Disorders - fifth edition [40]. Anxiety symptoms were measured by the 4-item PROMIS Emotional Distress-Anxiety, Short Form [39]. This measure has demonstrated clinical validity in patients with chronic health conditions including cancer [41] as well as pediatric patients [42]. Depression symptoms were measured by the 4-item PROMIS Emotional Distress-Depression, Short Form [39], which captures self-reported depression symptoms, and performs similar to legacy measures (Beck Depression Inventory, Center for Epidemiological Studies Depression) in adults diagnosed with cancer [43]. Both PROMIS measures asked participants to indicate the frequency of symptoms over the past 7 days on a five-point response scale (0-4). Item scores were summed to obtain the total raw score, which was then converted to a *T* score (mean 50, SD 10). The ranges are as follows: anxiety: *t*=40.3-81.6 and depression: *t*=41.0-79.4. The cutoffs on both scales are as follows: mild, *t*=55; moderate, *t*=60; and severe, *t*=70 [44].

##### Positive and Negative Emotions

Two-week retrospective reports of positive and negative emotions were measured with the modified version of the Differential Emotions Scale, which includes additional items to measure positive emotion [45]. Positive emotions included 10 items scored from 0 (“not at all”) to 4 (“most of the time”), with ranges from 0 to 40. Negative emotions included 9 items scored similarly (range: 0-36). Additionally, daily prompts for emotion ratings were triggered in the chatbot in both the experimental and control conditions. Six discrete emotions (happy, excited, content, worried, irritable/angry, and sad) were rated on a scale from 0 (“not at all”) to 8 (“extremely”). Multimedia Appendix 3 presents a depiction of this measure. Responses from the three positive and three negative emotion items were each averaged to make a single positive and negative emotion score (range: 0-24) for each time period of interest (daily for daily prompts and at each 2- and 4-week surveys).

#### Demographics

Demographics included year of birth, gender, year of cancer diagnosis, year of treatment completion, treatment institution, basic work/school status (full-time, part-time, none), ethnicity, highest level of education, and current city of residence.

#### Analyses

##### Chatbot Engagement

Means and SDs were reported for time spent on all sessions. Within the experimental group, means and SDs for chatbot feedback were reported based on the measures described above. Open-ended feedback was evaluated, and quotes were selected to exemplify prominent themes.

##### Well-Being Outcomes

Well-being outcomes were assessed using a series of multilevel mixed-effects linear models for intention to treat. Multilevel models were used because they accommodate missing data and nonindependence in observations. For each construct (anxiety, depression, positive/negative emotions), a separate mixed-effects model was used to evaluate the difference in magnitude of change from baseline to week 4 follow-up assessment as a function of intervention condition (experimental vs control) and the interaction of time by condition. Each model was evaluated on the basis of statistical significance (*P*<.05) of the interaction term. Given the pilot nature of the trial, in cases in which the *P* value approached significance, effect sizes were also examined for strength and clinically meaningful differences in main outcomes across groups.

To validate results, we separately modeled changes in primary outcomes within the control condition only to examine changes in primary outcomes from the 4-week survey (when the control condition received access to the intervention) to the 8-week follow-up (which allowed for a possible 4 week intervention window, similar to that of the experimental group). This model did not include any condition or interaction term.

To examine the relationship between chatbot engagement and well-being outcomes, we used mixed-effects models to separately model each outcome as a function of the number of engaged sessions between baseline and follow-up, intervention condition (experimental vs control), and the interaction of engaged sessions and condition.

## Results

### Retention

In total, 51 participants completed a baseline assessment and were randomized to a study condition (experimental group: 25; control group: 26; Figure 1). After excluding 6 control participants who were erroneously given access to chatbot content at 2 weeks instead of 4 weeks, the final analytic sample comprised 45 participants. The rate of follow-up survey completion was 73% (33/45) at 2 weeks, 73% (33/45) at 4 weeks, and 58% (26/45) at 8 weeks. We noted differences by group, with higher rates of survey completion in the control group, reaching statistical significance in week 8 (2 weeks: 80% in the control group, 68% in the experimental group, χ^2^: 0.8, *P*=.37; 4 weeks: 85% in the control group, 64% in the experimental group, χ^2^: 2.5, *P*=.12; 8 weeks: 75% in the control group, 44% in the experimental group, χ^2^: 4.28, *P*=.04).

### Participant Characteristics

Demographic information for the 45 participants included in the final analysis is presented in Table 1. Participants were mostly female (36/45, 80%), with an average age of 25 (SD 2.9; range: 19-29 years). Participants were on average 2.7 (SD 2.0) years postdiagnosis and 1.6 (SD 1.3) years postcompletion of active cancer treatment.

**Figure 1:**
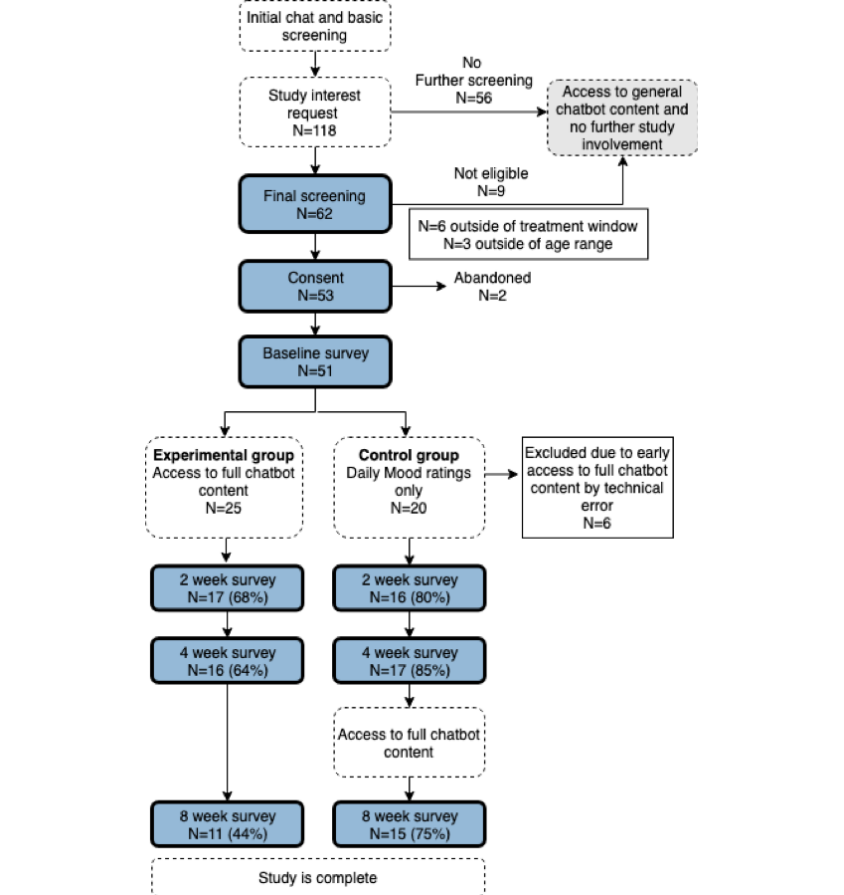
Participant recruitment and flow through the Vivibot pilot trial.

**Table 1:** Demographic data of participants.

Demographic		Experimental (n=25)	Control (n=20)	Total (N=45)
Age (years), mean (SD)		25 (3.1)	25 (2.9)	25 (2.9)
**Gender, n (%)**				
	Male	4 (16)	5 (25)	9 (20)
	Female	21 (84)	15 (75)	36 (80)
**Education, n (%)**				
	Less than high school	0 (0)	2 (10)	2 (4)
	High school graduate/General Educational Development	3 (12)	2 (10)	5 (11)
	Some college	8 (32)	9 (45)	17 (38)
	2-year college degree	2 (8)	1 (5)	3 (7)
	4-year college degree	10 (40)	5 (25)	15 (33)
	Master’s degree	2 (8)	1 (5)	3 (7)
**School/work status, n (%)**				
	I’m still figuring out my next move	5 (20)	3 (15)	8 (18)
	I’m going to school and/or work part time	9 (36)	4 (20)	13 (29)
	I’m going to school and/or work full time	11 (44)	13 (65)	24 (53)
**Ethnicity, n (%)**				
	White/Caucasian	19 (76)	18 (90)	37 (82)
	Hispanic or Latino	1 (4)	2 (10)	3 (7)
	Black or African American	3 (12)	0 (0)	3 (7)
	Asian or Pacific Islander	1 (4)	0 (0)	1 (2)
	Prefer not to answer	1 (4)	0 (0)	1 (2)
**Treatment history, mean (SD)**				
	Years postdiagnosis	2.7 (1.8)	2.8 (2.3)	2.7 (2.0)
	Years posttreatment	1.5 (1.4)	1.8 (1.2)	1.6 (1.3)

### Chatbot Engagement

During the 4 active weeks of the study, the experimental group spent an average of 73.8 (SD 52) min across an average of 12.1 (SD 7.1) engaged sessions chatting with *Vivibot*. The control group spent an average of 27.13 (SD 15.8) min across an average of 18.1 (SD 8.6) engaged sessions completing the 6-item emotion ratings only.

### Perceived Helpfulness and Open-Ended Feedback.

On average, participants in the experimental group rated their experience of chatting with *Vivibot* as helpful, with an average rating of 2.03/3 (SD 0.72; range: 0-3). They were also likely to recommend *Vivibot* to a friend, with an average rating of 6.9/10 (SD 2.6; range: 0-10). When asked why they were likely or unlikely to recommend *Vivibot* to a friend, participants remarked on the utility and nonjudgmental nature of talking to an automated agent:

When going through treatment it was hard not to bum out my friends talking about treatments and life. My whole perspective changed. And this is a way to openly talk about those changes and you present great paths to take those thoughts rather than trying to internalize or face those awkward conversations with healthy friends.

Additional themes related to having a shared experience with others who had undergone cancer treatment and being able to just “vent”:

Because as weird as it is talking to a robot, it’s nice to vent and be able to see others with cancer talking and speaking out about how they coped or felt during their treatment. Seeing that I’m not alone and having someone guide me to find the positives in my life now is really helpful.

Participants also particularly enjoyed the positive psychology content itself:

You give me new perspectives on things and help me set goals for myself and find things to be thankful for.

I also like the lessons you share…like looking for the good in bad situations or setting the goals to do random acts of kindness.

Participants who gave lower ratings of *Vivibot* were less specific in their feedback:

I just haven’t found it very helpful.

This bot kind of makes me feel like I’m being talked at rather than talking with.

Vivbot is annoying.

Full quotes from participants can be found in Multimedia Appendix 5.

### Well-Being Outcomes

#### Anxiety and Depression Symptoms

At baseline, both experimental and control groups presented with moderate levels of anxiety (experimental group: mean 64.5 [SD 6.1]; control group: mean 62.6 [SD 7.9]; threshold=60) and mild levels of depression (experimental group: mean 60.1 [SD 7.4]; control group: mean 59.0 [SD 9.2]; threshold=60).

Participants in the experimental group reported a greater reduction in anxiety than the control group at a trend level of statistical significance and small-to-moderate effect size (experimental reduction of 2.58 t-score units vs control of 0.7 units; z=–1.70; Cohen *d*=–0.41; *P*=.09; Table 2). Both experimental and control groups showed slight decreases in depressive symptom ratings with no evidence of a condition by time interaction (experimental reduction=1.83; control reduction=1.38; z=0.30; Cohen *d*=0.09; *P*=.77).

To validate the results, a post hoc analysis of the change between 4 weeks and 8 weeks in the control group (N=17), after receiving access to the full 28-day chatbot content, revealed a similar magnitude drop in anxiety symptoms (2.72 standardized points) and a statistical trend (*P*=.13). As in the experimental group, there was no significant reduction in depression in this subsample (Multimedia Appendix 4 presents for full results at 8 weeks).

#### Anxiety Symptoms by Engagement

When using engaged sessions to predict anxiety outcomes (as opposed to timepoint alone), there was a stronger trend-level relationship for group by engaged sessions interaction (z=–1.9, *P*=.06). There was no such trend for depression outcomes (z=–0.60, *P*=.55).

#### Positive and Negative Emotions

Both the experimental group and the control group showed a similar magnitude decrease in negative emotion in retrospective emotion ratings from baseline to week 4 (mean difference: experimental group: –0.31, control group: –0.23), with no significant or trend-level interaction by group for negative emotion at 4 weeks (z=0.23; Cohen *d*=–0.01; *P*=.97). This pattern was also reflected in the daily emotion ratings recorded in the chatbot, which showed a significant decrease in negative emotion reporting (z=–2.44; *P*=.02), but no significant interaction between groups (z=–0.74; *P*=.46).

Both the experimental group and control group showed almost no change in retrospective positive emotion ratings from baseline to week 4 (mean difference: experimental group: 0.04, control group: –0.08). Interestingly, the daily emotion ratings reported directly in the chatbot showed a significant main effect of time for increased positive emotion across groups (z=3.56; *P*=.002). Further, there was a significant interaction by group (z=–2.07; *P*=.04); however, this effect was in the opposite direction from our hypothesis, as the control group showed greater increases in positive daily emotion ratings reported directly in the chatbot, compared to the experimental group.

**Table 2:** Results for well-being outcomes (anxiety, depression, positive emotion, and negative emotion) across conditions and for experimental by control interactions.

Condition		Baseline, mean (SD)	Week 4, mean (SD)	Difference of means	Interaction effect size		*P* value	
**Anxiety**						–0.41		.09
	Experimental	64.5 (6.1)	61.9 (7.7)	–2.58				
	Control	62.6 (7.9)	63.3 (5.5)	0.7				
**Depression**						0.09		.77
	Experimental	60.1 (7.4)	58.2 (8.8)	–1.83				
	Control	59.0 (9.2)	57.7 (6.1)	–1.38				
**Negative emotion**						–0.01		.97
	Experimental	1.8 (0.7)	1.5 (0.9)	–0.31				
	Control	1.9 (0.7)	1.6 (0.6)	–0.23				
**Positive emotion**						0.07		.82
	Experimental	2.4 (0.8)	2.5 (1.0)	0.04				
	Control	2.3 (0.9)	2.3 (0.8)	–0.08				

## Discussion

### Principal Findings

High engagement and positive ratings of *Vivibot* suggest that a chatbot provides a useful and acceptable format for young adults to connect with a positive psychology intervention. Qualitative responses supplemented findings from traditional metrics of user engagement such as the number of times *Vivibot* was accessed and the length of time in sessions. Feedback indicated an overall positive response to the chatbot and guided developers to generate product improvements on specific features and content once the study was completed. Further development of this tool and potentially combining it with other person-to-person psychosocial interventions may enhance engagement even further.

Overall, positive psychology skills, delivered by a chatbot, were perceived as helpful and nonjudgmental by young adults who had undergone cancer treatment. Positive emotion, when stimulated through skills-based interventions, is thought to influence health outcomes, both directly and through the mediating influence of factors including changing health behaviors, improving physiological functioning, and increasing resources that influence health [15,46]. Possible mechanisms through which a positive psychology intervention could be influencing anxiety in young people who have undergone cancer treatment include increasing feelings of support and social control, which are influenced by positive emotion [47] and potentially poor after cancer treatment. Talking to a nonjudgmental “robot” could also have increased participants’ receptivity to learn skills to manage stress, which is another core mediator of the health effects of positive emotion [15]. Additional work should be done to understand the effects of individual positive emotion intervention skills and to link skill building to subsequent long-term health impacts. The experience of a bot-based intervention as nonjudgmental shows promise for future study using this format with young people.

Based on previous work with positive psychology interventions [46], we hypothesized that engagement with *Vivibot* would result in improved psychological well-being in the form of reduced depression and anxiety symptoms and more positive and fewer negative emotions. Engagement with *Vivibot* was associated with a reduction in anxiety symptoms. Although not significant, the effect size for the main anxiety analysis of .41 in this pilot trial was small to moderate by Cohen [48] standards. Further, it was comparable to that found in a meta-analysis of psychosocial support for cancer (effect size=0.42) [49] and a positive psychology intervention for caregivers (effect size=0.33) [50]. After converting to a common scale (Multimedia Appendix 6), the change we saw in the General Anxiety Disorder-7 items equivalent scores from baseline to 4 weeks in the treatment group (reduction of 1.82 points) was greater than that found in trials testing the Woebot chatbot (reduction of 0.7 points over 2 weeks [35]) and the X2AI Artificial Intelligence tool *Tess* (reduction of 1.4 points over 4 weeks) [51]. Thus, *Vivibot* is associated with, at a minimum, a comparable reduction in anxiety symptoms as other psychosocial interventions for people with cancer and similar digital health tools available on the market for general populations.

Among those who used *Vivibot*, depression symptoms were not reduced compared to those who only completed daily emotion ratings. The active nature of the control condition in this trial could have accounted for this. The emotion rating control on its own appeared to carry some benefits for participants (rating emotions was associated with decreased negative emotion and increased positive emotion). Additionally, anecdotally, some users remarked on how completing the emotion ratings was helpful to them when asked why they would be willing to refer *Vivibot* to a friend (eg, “This is helpful to review your feelings” and “I like the daily check in questions because it reminds me to check in with myself on how I am feeling and think about why my answers might change from day to day;” Multimedia Appendix 5). This is consistent with the literature showing that emotion labeling itself can contribute to beneficial emotion regulation in response to negative events [52]. Of note, the depression symptom reduction, when converted to the Patient Health Questionnaire - 9 items equivalent, in the experimental group in our trial (1.5 points over 4 weeks) was comparable to that reported in the trial of the X2AI tool (0.9 points over 4 weeks) [51]. Since both the experimental group and the control group experienced the same emotion rating exercise, it is possible that the emotion ratings themselves carried the benefit of reductions in negative emotion, increases in positive emotion, and reductions in depression reported here in both experimental and control conditions. Further studies using a control group that did not engage in emotion labeling would be needed to more fully identify the mechanisms behind these effects.

This work extends findings showing promise for technology-enabled solutions to address mental health problems. Internet-based interventions have been shown to work at least as effectively as face-to-face intervention in improving mental health across a range of conditions. In addition to opportunities to scale interventions, online delivery offers self-pacing and the ability to incorporate practices into daily life, thereby transcending geography, time, and space [53]. Our work further suggests the chatbot interface, which is less costly than a human-delivered intervention, is a promising format to deliver psychosocial interventions to young adults. *Vivibot* included many features found to be associated with positive outcomes in internet-based interventions for depression among adolescents, including surface credibility, esthetics, and content appeal to the specific population; appropriate reduction of content compared to that in traditional interventions; and use of self-monitoring [54]. Acceptance of this positive psychology intervention is therefore not surprisingly consistent with the broad acceptance found for online-based interventions for depression and anxiety among adolescents [55].

Further, this work contributes to the growing body of literature showing promise for interventions delivered via social media in supporting short-term symptom improvement and behavior change among young adults. Most work in this area has focused on health behaviors such as smoking cessation [56] and physical activity among young people with cancer [57]; this study provides early evidence that social media–mediated intervention can be harnessed to reduce mental health symptoms. Additional randomized trials are needed to strengthen conclusions for this particular intervention and for this field in a broad sense.

### Limitations

There are several limitations to this study that weaken the generalizability of these findings and motivate further investigations. First, this was a small pilot study that was not powered to detect significant effects in the psychological outcomes measured. Due to the relatively small population size and heterogeneity of treatment facilities, it is difficult to identify and recruit adolescents and young adults who had completed treatment for cancer for research studies. Our results need to be replicated in a sample large enough to reliably detect a moderate-sized effect between groups. Second, this study was limited to participants willing and able to access *Vivibot* via Facebook Messenger. Although Facebook Messenger had the advantage of being widely accessible to this population and has almost full saturation among young adults in the United States [58], it is possible that participants did not wish to engage with Facebook Messenger in light of the controversy surrounding the privacy policies of Facebook during the study period. Other platforms, including WhatsApp and smartphone apps, should be considered in future adaptations of *Vivibot*. A third limitation was that neither the type nor stage of cancer was considered in the analyses. Due to the unique developmental and psychosocial needs of young people who have been treated for cancer, this investigation collapsed across cancer diagnoses to focus on a more targeted age range. It is possible that patients with different types of cancers would react differently to the intervention content of *Vivibot*; future work with a larger sample size should investigate this aspect further.

### Conclusions

The *Vivibot* chatbot was engaging to users and resulted in anxiety reduction comparable to that found in the literature with digital health interventions targeting anxiety and depression. Given their short duration, non–artificial intelligence-based content-delivery system results, and ease with which it could be delivered in Facebook Messenger or other chat platforms, chatbots are promising for supporting the mental health needs of young people with cancer at a vulnerable time in their lives. The randomized design of this trial extends the promising findings of other positive psychology interventions delivered online [59] and gives confidence that the effect size seen with anxiety is worthy of follow-up. In addition to a larger trial, future research should consider whether the tool is as effective when delivered to other populations or platforms.

## Appendix

Multimedia Appendix 1 Vivibot content overview.

Multimedia Appendix 2 Example user experience of positive psychology skill.

Multimedia Appendix 3 Screen shot of daily mood ratings.

Multimedia Appendix 4 Comparisons for all outcomes at 8 weeks.

Multimedia Appendix 5 Recommendation ratings.

Multimedia Appendix 6 Converted scores on the PROMIS-Anxiety and PROMIS-Depression scales.

Multimedia Appendix 7 CONSORT‐EHEALTH checklist (V 1.6.1).

## Abbreviations

PRISM: Promoting Resilience in Stress Management
PROMIS: Patient-Reported Outcomes Measurement Information System
UCLA: University of California, Los Angeles
