# Supplementary material for: Use of the Chatbot “Vivibot” to Deliver Positive Psychology Skills and Promote Well-Being Among Young People After Cancer Treatment: Randomized Controlled Feasibility Trial
Source: JMIR Mhealth Uhealth. 2019 Oct 31;7(10):e15018. doi: 10.2196/15018 (PMC6913733; doi:10.2196/15018)

Supplement 1. Vivibot Content Overview

As illustrated in the diagram below, each daily chat with Vivibot followed the same basic structure. First the user would receive a notification with a basic greeting (for example: "Hey, its me, your friendly neighborhood bot!") which the user could respond to if they wanted to chat. If the user was willing to engage then they began the session by rating their current emotional state on a 6-item scale (happy, content, excited, sad, irritable/angry and worried). After completing the emotion scale, participants were guided through a daily lesson in positive psychology. These lessons were randomized across participants and across sessions such that each individual received a different order and set of content. Each for the seven skill topics included a teaching lesson the first time the user encounters that skill to introduce the user to the skill and allow guided reflection. On subsequent encounters of that skill, the user would receive a "practice" script that focused on guided reflection only. After the completion of the skill, the user was given an opportunity to "Vent" or free write what was on their mind, followed by an option to see an additional video of a cancer survivor story before final conversation sign off. Users were only allowed to complete one lesson per day and were given an automated message to return the following day if they tried to continue engaging.

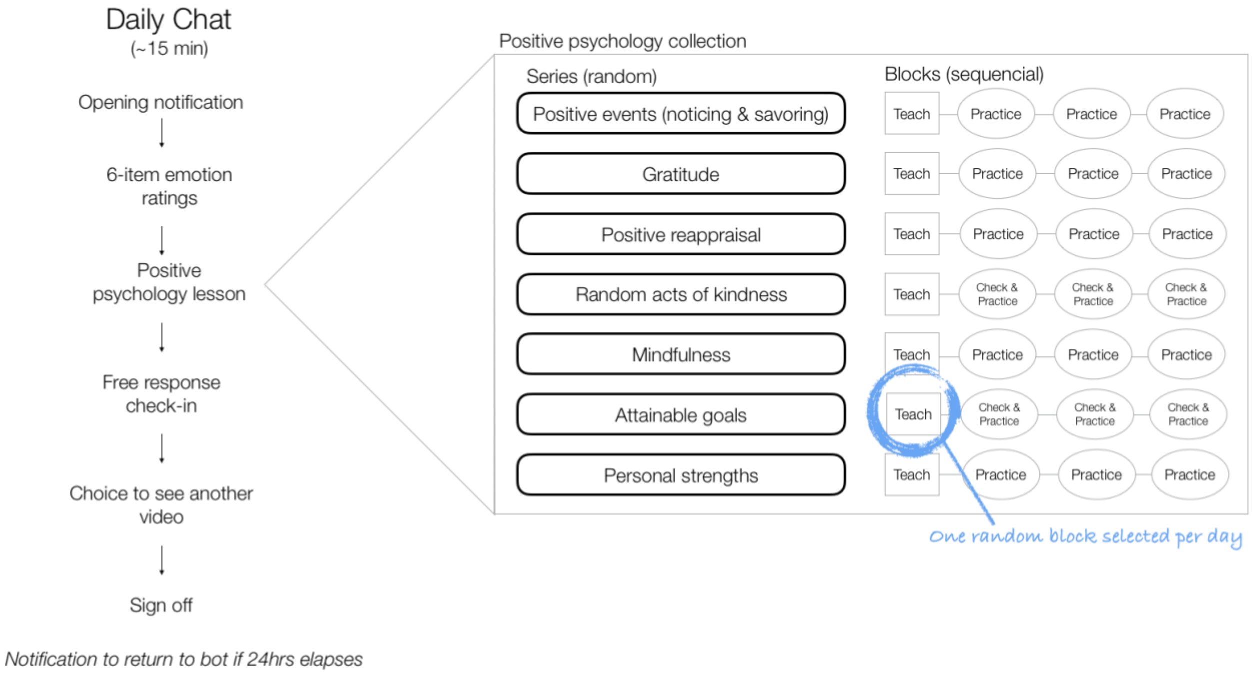

Supplement: Multimedia Appendix 1 [file mhealth_v7i10e15018_app1.pdf]
