# Supplementary material for: Use of the Chatbot “Vivibot” to Deliver Positive Psychology Skills and Promote Well-Being Among Young People After Cancer Treatment: Randomized Controlled Feasibility Trial
Source: JMIR Mhealth Uhealth. 2019 Oct 31;7(10):e15018. doi: 10.2196/15018 (PMC6913733; doi:10.2196/15018)
Supplement: Multimedia Appendix 2 [file mhealth_v7i10e15018_app2.pdf]

Supplement 2. Example user experience of positive psychology skill.

The attached video file “Vivibot_skill_demo_reappraisal.mp4” shows an example of a daily chat that includes the skill Positive Reappraisal. Here is the full written version of this lesson:

Bot:	Oh, hey there. I was having a shit day so I'm watching this video of my friend Lexus. Wanna join? I think you'll relate	
**User:**	**sure**	
Bot:	[SURVIVOR VIDEO]	
	I love how she says finds the "beautiful in this mess." Even though she's angry - and she's dealing with some pretty ugly situations, health-wise - she always sees the beauty ... of the people helping her and wanting her to persevere.	
Bot:	Isn't SHE beautiful?	
**User:**	💖	
Bot:	Her ability to turn legit lemons into some sweetass lemonade is something psychologists call "positive reappraisal."	
Bot:	a.k.a. "finding the silver lining" or "looking on the bright side," if you're a total cheezball like me 😎	
**User:**	**gotcha**	
Bot:	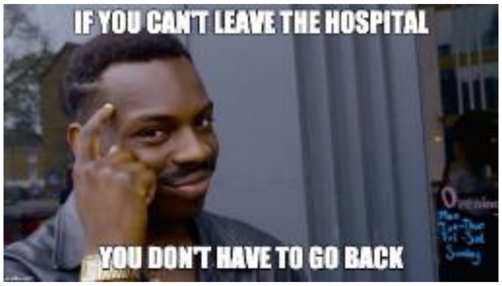	
Bot:	see what I mean	
**User:**	**Lol**	
Bot:	I know what you may be thinking:	

Bot:	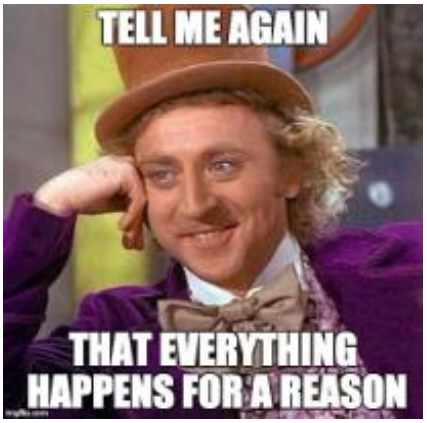	
Bot:	it's for sure annoying when people force positivity on you and try to spin having cancer as some sort of fateful journey	
**User:**	**ugh the j word**	**sure**
Bot:	But learning to think differently about your own situation can for real be helpful. let's give it a quick try?	
**User:**	**sure**	**if you say so**
Bot:	Okay! Maybe you had a specific moment during cancer treatment that made you angry. Like someone took 'your' chemo chair... or your favorite nurse was with a different patient one day...	
**User:**	**Yup**	**Something like that**
Bot:	what happened?	
**User:**	**[FREE RESPONSE]**	
Bot:	how did you feel? I mean ... not great, ovi	
**User:**	**[FREE RESPONSE]**	
Bot:	Ok, now ... put on your Lexus goggles: Can you spot a flash of beauty in that particular shitfest? Like maybe having to sit in a new chemo chair gave you a bomb ocean view or your new nurse ended up turning you on to a great band. Anything like that?	
**User:**	**Yeah...**	**Hmm...not really**
Bot:	What was it?	OK, well if you were FORCED by a charming but persistent robot to think of one positive thing - however small - about that moment, what would it be?
**User:**	**[FREE RESPONSE]**	**[FREE RESPONSE]**

Bot:	nice ... wait, is your name, Lexus!? 🌞	nice ... I knew you could do it. 👊
Bot:	Now think of an example from the last few days ... where you reacted less than positively in an annoying situation. What happened?	
**User:**	**[FREE RESPONSE]**	
Bot:	How could you flip that narrative to make it a positive story?	
**User:**	**[FREE RESPONSE]**	**<>**
	Well done you. And, hey, if you have a meltdown once in a while, it's ok. You're human. I meltdown sometimes and I don't even have real emotions. [illegible]	
**User:**	**Team meltdown**	**Always composed**
Bot:	But, seriously, try flipping the script in your head to focus on the good stuff whenever you can. It's a powerful Jedi mindtrick ...	
	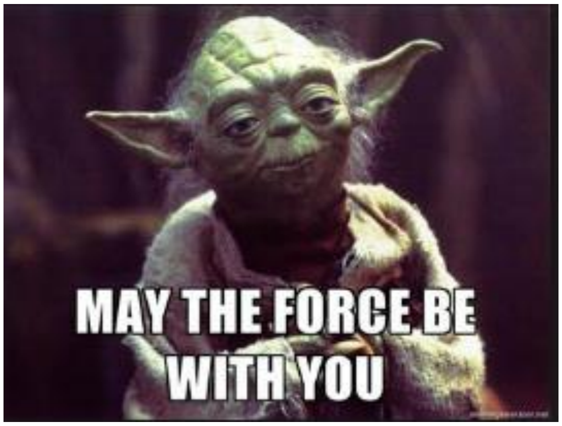	
Bot:	Rock on.	
