# Supplementary figures and images for: Use of the Chatbot “Vivibot” to Deliver Positive Psychology Skills and Promote Well-Being Among Young People After Cancer Treatment: Randomized Controlled Feasibility Trial
Source: JMIR Mhealth Uhealth. 2019 Oct 31;7(10):e15018. doi: 10.2196/15018 (PMC6913733; doi:10.2196/15018)

### Supplement 3. Screen shot of daily mood ratings

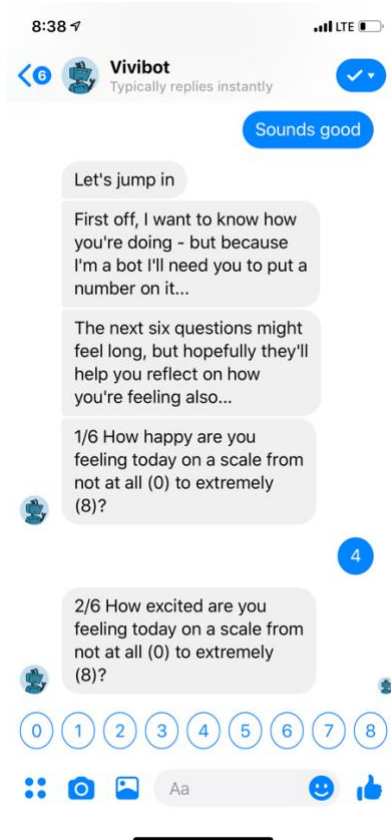

Supplement: Multimedia Appendix 3 [file mhealth_v7i10e15018_app3.pdf]
