# Supplementary material for: Use of the Chatbot “Vivibot” to Deliver Positive Psychology Skills and Promote Well-Being Among Young People After Cancer Treatment: Randomized Controlled Feasibility Trial
Source: JMIR Mhealth Uhealth. 2019 Oct 31;7(10):e15018. doi: 10.2196/15018 (PMC6913733; doi:10.2196/15018)
Supplement: Multimedia Appendix 4 [file mhealth_v7i10e15018_app4.pdf]

Supplement 4. Comparisons for all outcomes at 8 weeks

	Condition	Baseline M (Stdev)	Week 4 M (Stdev)	Difference from baseline	Change from baseline	Week 8 M (Stdev)	Difference from baseline	Change from baseline	Difference from week4	Change from week4
**Anxiety**	Experimental	64.5 (6.1)	61.9 (7.7)	-2.58	**p=0.03**	63.3 (6.2)	-1.23	p=0.41	1.34	p=0.59
	Control	62.6 (7.9)	63.3 (5.5)	0.7	p=0.70	60.6 (9.6)	-2.02	p=0.34	-2.72	**p=0.13**
**Depression**	Experimental	60.1 (7.4)	58.2 (8.8)	-1.83	p=0.50	57.3 (10.1)	-2.76	p=0.31	-0.93	p=0.61
	Control	59.0 (9.2)	57.7 (6.1)	-1.38	p=0.30	58.0 (7.7)	-1.09	p=0.65	0.29	p=0.70
**Negative E**	Experimental	1.8 (0.7)	1.5 (0.9)	-0.31	**p=0.12**	1.4 (0.7)	-0.43	**p=0.03**	-0.12	p=0.29
	Control	1.9 (0.7)	1.6 (0.6)	-0.23	**p=0.15**	1.6 (0.6)	-0.27	p=0.22	-0.04	p=0.85
**Positive E**	Experimental	2.4 (0.8)	2.5 (1.0)	0.04	p=0.91	2.5 (0.8)	0.05	p=0.87	0.01	p=0.89
	Control	2.3 (0.9)	2.3 (0.8)	-0.08	p=0.82	2.4 (0.8)	0.09	p=0.70	0.16	p=0.53
