# Supplementary material for: Use of the Chatbot “Vivibot” to Deliver Positive Psychology Skills and Promote Well-Being Among Young People After Cancer Treatment: Randomized Controlled Feasibility Trial
Source: JMIR Mhealth Uhealth. 2019 Oct 31;7(10):e15018. doi: 10.2196/15018 (PMC6913733; doi:10.2196/15018)
Supplement: Multimedia Appendix 5 [file mhealth_v7i10e15018_app5.pdf]

Supplement 5. Recommendation ratings

On the seventh engaged session, users were asked “How likely would you be to recommend me to a friend? (0 means not at all likely and 10 means extremely likely)” and after giving the score “Could you tell me a bit about why you gave that score?”. Control participants were only asked this question on their 7th engaged session after receiving full chatbot content (they were not asked this question at all during the period when they received emotion ratings only). Note that control* indicates control participants who received full chatbot access early (after 2-weeks) and were therefor not included in the primary analysis.

Group	Score	Reason for Score
Experimental	10	When going through treatment it was hard not to bum out my friends talking about treatments and life. My whole perspective changed. And this is a way to openly talk about those changes and you present great paths to take those thoughts rather than trying to internalize or face those awkward conversations with healthy friends
Experimental	10	This is helpful to review your feelings. Usually going through treatment there isn't a lot of time to assess how you feel in "survival" mode. Nor after when you just want to be "normal".
Control*	10	I like the language that the bot uses. I think it will resonate well with the younger target demographic.
Control*	10	It’s nice to have this as a resource
Control*	10	I think having a outlet to talk to that's specifically cancer geared will have a tremendously positive impact.
Control	10	Yr a good support
Experimental	9	Because like I said you give me new perspectives on things and help me set goals for myself and find things to be thankful for
Experimental	9	Very informative advice, not too annoying, and I like that the bot is a girl lol
Control	9	It gave me perspective and thought about the feelings of others and solutions to some of my problems and things I can do to help the way I feel
Experimental	8	I also like the lessons you share. And would even appreciate getting reminders throughout the week of things I told you i'd do like looking for the good in bad situations or setting the goals to do random acts of kindness.
Experimental	8	It's nice to have someone/thing check in on you every day but sometimes it's frustrating/upsetting when I have to rate my day at the beginning of each conversation
Control	8	If I had more young friends struggling with cancer this would've gotten a higher score the videos are nice and it's nice to reflect on my emotional state
Control	8	I definitely would recommend to a friend i didnt put ten because i dont know many cancer survivors/patients and because some people wouldn't put the work in to get the benefits of the app
Control	8	There is some room for improvement, but I think it has really helped it!
Experimental	7	I don't have many friends going through cancer right now (thank goodness lol) so I can't really recommend to them. Could be cool to add a section for friends of survivors and how to communicate and help through the process.
Experimental	7	It may be because I’m talking to you everyday, but the “how are you feeling” with the same questions each time we talk is repetitively. What follows is great tho!

Experimental	7	Because as weird as it is talking to a robot, it's nice to vent and be able to see others with cancer talking and speaking out about how they coped or felt during their treatment. Seeing that I'm not alone and having someone guide me to find the positives in my life now is really helpful. I think it could use some work but have overall been pleased with it
Experimental	7	Im not sure if this would help someone to have company during cancer treatment or not. I was literally too tired to function- or think to send messages.
Control*	7	I like the daily check in questions because it reminds me to check in with myself on how I am feeling and think about why my answers might change from day to day. However I sometimes don't want to do more than that. There have been a few lessons that were long and required more interaction than I wanted to give at that time. Overall thought I have enjoyed it
Control*	7	Well it depends on the friend but I think you could be very helpful to people who are neutropenic. Or like me who are just anxious and need to talk. I like you a lot but wish there were a few more options
Experimental	6	It's hard to communicate with a bot. But at the same time I'd rather know I'd not have to really give much effort if I was just looking for the idea of someone being there
Experimental	6	It's helpful as a survivor but isn't very tailored to my specific worries and thoughts
Experimental	6	its nice to have someone check in on you daily and have something to go to bout cancer, but the video 'experts' need some work and also theres the limitations of the responses that are able to be understood.
Control	6	Idk
Control	5	Sometimes I don't need assistance
Control	5	i don't really get much out of it. i do like the videos though. it doesn't feel personal or sincere when you're talking to a robot
Experimental	3	This bot kind of makes me feel like I'm being talked at rather than talking with
Control	3	I just haven't found it very helpful.
Experimental	0	Vivbot is annoying
