# Supplementary material for: Use of the Chatbot “Vivibot” to Deliver Positive Psychology Skills and Promote Well-Being Among Young People After Cancer Treatment: Randomized Controlled Feasibility Trial
Source: JMIR Mhealth Uhealth. 2019 Oct 31;7(10):e15018. doi: 10.2196/15018 (PMC6913733; doi:10.2196/15018)
Supplement: Multimedia Appendix 6 [file mhealth_v7i10e15018_app6.pdf]

Supplement 6. Converted scores on the PROMIS-Anxiety and PROMIS-Depression scales

To facilitate comparison of results to previous reports, PROMIS scores for each individual were converted to PHQ9 and GAD7 scores using previously published conversation tables.

	Condition	Baseline M (Stdev)	Week 4 M (Stdev)	Difference of the means
**Anxiety (GAD7 Converted)**	Experimental	11.6 (4.2)	9.8 (4.7)	-1.82
**Anxiety (GAD7 Converted)**	Control	10.5 (5.0)	10.9 (3.6)	0.38
**Depression (PHQ9 Converted)**	Experimental	10.8 (5.2)	9.5 (6.2)	-1.31
**Depression (PHQ9 Converted)**	Control	10.3 (6.2)	8.8 (3.8)	-1.48

GAD7 table: https://www.ncbi.nlm.nih.gov/pmc/articles/PMC4046852/pdf/nihms564318.pdf

PHQ9 conversion table: https://www.ncbi.nlm.nih.gov/pmc/articles/PMC5515387/
