# Supplementary material for: Use of the Chatbot “Vivibot” to Deliver Positive Psychology Skills and Promote Well-Being Among Young People After Cancer Treatment: Randomized Controlled Feasibility Trial
Source: JMIR Mhealth Uhealth. 2019 Oct 31;7(10):e15018. doi: 10.2196/15018 (PMC6913733; doi:10.2196/15018)
Supplement: Multimedia Appendix 7 [file mhealth_v7i10e15018_app7.pdf]

CONSORT-EHEALTH (V 1.6.1) - Submission/Publication Form

The CONSORT-EHEALTH checklist is intended for authors of randomized trials evaluating web-based and Internet-based applications/interventions, including mobile interventions, electronic games (incl multiplayer games), social media, certain telehealth applications, and other interactive and/or networked electronic applications. Some of the items (e.g. all subitems under item 5 - description of the intervention) may also be applicable for other study designs.

The goal of the CONSORT EHEALTH checklist and guideline is to be
a) a guide for reporting for authors of RCTs,
b) to form a basis for appraisal of an ehealth trial (in terms of validity)

CONSORT-EHEALTH items/subitems are MANDATORY reporting items for studies published in the Journal of Medical Internet Research and other journals / scientific societies endorsing the checklist.

Items numbered 1., 2., 3., 4a., 4b etc are original CONSORT or CONSORT-NPT (non-pharmacologic treatment) items.
Items with Roman numerals (i., ii, iii, iv etc.) are CONSORT-EHEALTH extensions/clarifications.

As the CONSORT-EHEALTH checklist is still considered in a formative stage, we would ask that you also RATE ON A SCALE OF 1-5 how important/useful you feel each item is FOR THE PURPOSE OF THE CHECKLIST and reporting guideline (optional).

Mandatory reporting items are marked with a red *.
In the textboxes, either copy & paste the relevant sections from your manuscript into this form - please include any quotes from your manuscript in QUOTATION MARKS,
or answer directly by providing additional information not in the manuscript, or elaborating on why the item was not relevant for this study.

YOUR ANSWERS WILL BE PUBLISHED AS A SUPPLEMENTARY FILE TO YOUR PUBLICATION IN JMIR AND ARE CONSIDERED PART OF YOUR PUBLICATION (IF ACCEPTED).
Please fill in these questions diligently. Information will not be copyedited, so please use proper spelling and grammar, use correct capitalization, and avoid abbreviations.

DO NOT FORGET TO SAVE AS PDF _AND_ CLICK THE SUBMIT BUTTON SO YOUR ANSWERS ARE IN OUR DATABASE !!!

Citation Suggestion (if you append the pdf as Appendix we suggest to cite this paper in the caption):
Eysenbach G, CONSORT-EHEALTH Group
CONSORT-EHEALTH: Improving and Standardizing Evaluation Reports of Web-based and Mobile Health Interventions
J Med Internet Res 2011;13(4):e126
URL: http://www.jmir.org/2011/4/e126/
doi: 10.2196/jmir.1923
PMID: [illegible]

PMID: 22209829

* Required

Your name *

First Last

Danielle Ramo

Primary Affiliation (short), City, Country *

University of Toronto, Toronto, Canada

Hopelab, San Francisco, CA

Your e-mail address *

abc@gmail.com

dramo@hopelab.org

Title of your manuscript *

Provide the (draft) title of your manuscript.

Use of the Chatbot “Vivibot” to Deliver Positive Psychology Skills and Promote Well-Being Among Young People After Cancer Treatment: Randomized Controlled Feasibility Trial

Name of your App/Software/Intervention *

If there is a short and a long/alternate name, write the short name first and add the long name in brackets.

Vivibot

Evaluated Version (if any)

e.g. "V1", "Release 2017-03-01", "Version 2.0.27913"

n/a

Language(s) *

What language is the intervention/app in? If multiple languages are available, separate by comma (e.g. "English, French")

English

URL of your Intervention Website or App

e.g. a direct link to the mobile app on app in appstore (itunes, Google Play), or URL of the website. If the intervention is a DVD or hardware, you can also link to an Amazon page.

https://grythealth.com/vivibot

URL of an image/screenshot (optional)

Your answer

Accessibility *

Can an enduser access the intervention presently?

- (selected) access is free and open
- access only for special usergroups, not open
- access is open to everyone, but requires payment/subscription/in-app purchases
- app/intervention no longer accessible
- Other:

Primary Medical Indication/Disease/Condition *

e.g. "Stress", "Diabetes", or define the target group in brackets after the condition, e.g. "Autism (Parents of children with)", "Alzheimers (Informal Caregivers of)"

Cancer (Young adults who have undergc

Primary Outcomes measured in trial *

comma-separated list of primary outcomes reported in the trial

engagement

Secondary/other outcomes

Are there any other outcomes the intervention is expected to affect?

depression symptoms, anxiety symptoms, positive emotions, negative emotions

Recommended "Dose" *

What do the instructions for users say on how often the app should be used?

- (selected) Approximately Daily
- Approximately Weekly
- Approximately Monthly
- Approximately Yearly
- "as needed"
- Other:

Approx. Percentage of Users (starters) still using the app as recommended after 3 months *

- (selected) unknown / not evaluated
- 0-10%
- 11-20%
- 21-30%
- 31-40%
- 41-50%
- 51-60%
- 61-70%
- 71%-80%
- 81-90%
- 91-100%
- Other:

Overall, was the app/intervention effective? *

- ◯ yes: all primary outcomes were significantly better in intervention group vs control
- ◯ partly: SOME primary outcomes were significantly better in intervention group vs control
- ◉ no statistically significant difference between control and intervention
- ◯ potentially harmful: control was significantly better than intervention in one or more outcomes
- ◯ inconclusive: more research is needed
- ◯ Other:

Article Preparation Status/Stage *

At which stage in your article preparation are you currently (at the time you fill in this form)

- ◯ not submitted yet - in early draft status
- ◯ not submitted yet - in late draft status, just before submission
- ◯ submitted to a journal but not reviewed yet
- ◯ submitted to a journal and after receiving initial reviewer comments
- ◉ submitted to a journal and accepted, but not published yet
- ◯ published
- ◯ Other:

Journal *

If you already know where you will submit this paper (or if it is already submitted), please provide the journal name (if it is not JMIR, provide the journal name under "other")

- ○ not submitted yet / unclear where I will submit this
- ○ Journal of Medical Internet Research (JMIR)
- ◉ JMIR mHealth and UHealth
- ○ JMIR Serious Games
- ○ JMIR Mental Health
- ○ JMIR Public Health
- ○ JMIR Formative Research
- ○ Other JMIR sister journal
- ○ Other:

Is this a full powered effectiveness trial or a pilot/feasibility trial? *

- ◉ Pilot/feasibility
- ○ Fully powered

Manuscript tracking number *

If this is a JMIR submission, please provide the manuscript tracking number under "other" (The ms tracking number can be found in the submission acknowledgement email, or when you login as author in JMIR. If the paper is already published in JMIR, then the ms tracking number is the four-digit number at the end of the DOI, to be found at the bottom of each published article in JMIR)

- ○ no ms number (yet) / not (yet) submitted to / published in JMIR

Other: #15018

TITLE AND ABSTRACT

1a) TITLE: Identification as a randomized trial in the title

1a) Does your paper address CONSORT item 1a? *

I.e does the title contain the phrase "Randomized Controlled Trial"? (if not, explain the reason under "other")

- (selected) yes
- Other:

1a-i) Identify the mode of delivery in the title

Identify the mode of delivery. Preferably use “web-based” and/or “mobile” and/or “electronic game” in the title. Avoid ambiguous terms like “online”, “virtual”, “interactive”. Use “Internet-based” only if Intervention includes non-web-based Internet components (e.g. email), use “computer-based” or “electronic” only if offline products are used. Use “virtual” only in the context of “virtual reality” (3-D worlds). Use “online” only in the context of “online support groups”. Complement or substitute product names with broader terms for the class of products (such as “mobile” or “smart phone” instead of “iphone”), especially if the application runs on different platforms.

	1	2	3	4	5	
subitem not at all important	○	○	○	○	●	essential

Does your paper address subitem 1a-i? *

Copy and paste relevant sections from manuscript title (include quotes in quotation marks "like this" to indicate direct quotes from your manuscript), or elaborate on this item by providing additional information not in the ms, or briefly explain why the item is not applicable/relevant for your study

Chatbot

1a-ii) Non-web-based components or important co-interventions in title

Mention non-web-based components or important co-interventions in title, if any (e.g., “with telephone support”).

	1	2	3	4	5	
subitem not at all important	(●)	( )	( )	( )	( )	essential

Does your paper address subitem 1a-ii?

Copy and paste relevant sections from manuscript title (include quotes in quotation marks "like this" to indicate direct quotes from your manuscript), or elaborate on this item by providing additional information not in the ms, or briefly explain why the item is not applicable/relevant for your study

n/a

1a-iii) Primary condition or target group in the title

Mention primary condition or target group in the title, if any (e.g., “for children with Type I Diabetes”) Example: A Web-based and Mobile Intervention with Telephone Support for Children with Type I Diabetes: Randomized Controlled Trial

	1	2	3	4	5	
subitem not at all important	( )	( )	( )	( )	(●)	essential

Does your paper address subitem 1a-iii? *

Copy and paste relevant sections from manuscript title (include quotes in quotation marks "like this" to indicate direct quotes from your manuscript), or elaborate on this item by providing additional information not in the ms, or briefly explain why the item is not applicable/relevant for your study

"Among Young People After Cancer Treatment"

1b) ABSTRACT: Structured summary of trial design, methods, results, and conclusions

NPT extension: Description of experimental treatment, comparator, care providers, centers, and blinding status.

1b-i) Key features/functionalities/components of the intervention and comparator in the METHODS section of the ABSTRACT

Mention key features/functionalities/components of the intervention and comparator in the abstract. If possible, also mention theories and principles used for designing the site. Keep in mind the needs of systematic reviewers and indexers by including important synonyms. (Note: Only report in the abstract what the main paper is reporting. If this information is missing from the main body of text, consider adding it)

	1	2	3	4	5	
subitem not at all important	○	○	○	○	◉	essential

Does your paper address subitem 1b-i? *

Copy and paste relevant sections from the manuscript abstract (include quotes in quotation marks "like this" to indicate direct quotes from your manuscript), or elaborate on this item by providing additional information not in the ms, or briefly explain why the item is not applicable/relevant for your study

"Participants were randomized to either immediate access to Vivibot content (experimental group) or access to only daily emotion ratings and access to full chatbot content after 4 weeks (control). Created using a human-centered design process with young adults treated for cancer, Vivibot content includes 4 weeks of positive psychology skills, daily emotion ratings, video, and other material produced by survivors, and periodic feedback check-ins."

1b-ii) Level of human involvement in the METHODS section of the ABSTRACT

Clarify the level of human involvement in the abstract, e.g., use phrases like “fully automated” vs. “therapist/nurse/care provider/physician-assisted” (mention number and expertise of providers involved, if any). (Note: Only report in the abstract what the main paper is reporting. If this information is missing from the main body of text, consider adding it)

	1	2	3	4	5	
subitem not at all important	◉	○	○	○	○	essential

Does your paper address subitem 1b-ii?

Copy and paste relevant sections from the manuscript abstract (include quotes in quotation marks "like this" to indicate direct quotes from your manuscript), or elaborate on this item by providing additional information not in the ms, or briefly explain why the item is not applicable/relevant for your study

n/a, there is no level of human involvement in the intervention - it is entirely automated

1b-iii) Open vs. closed, web-based (self-assessment) vs. face-to-face assessments in the METHODS section of the ABSTRACT

Mention how participants were recruited (online vs. offline), e.g., from an open access website or from a clinic or a closed online user group (closed usergroup trial), and clarify if this was a purely web-based trial, or there were face-to-face components (as part of the intervention or for assessment). Clearly say if outcomes were self-assessed through questionnaires (as common in web-based trials). Note: In traditional offline trials, an open trial (open-label trial) is a type of clinical trial in which both the researchers and participants know which treatment is being administered. To avoid confusion, use “blinded” or “unblinded” to indicated the level of blinding instead of “open”, as “open” in web-based trials usually refers to “open access” (i.e. participants can self-enrol). (Note: Only report in the abstract what the main paper is reporting. If this information is missing from the main body of text, consider adding it)

	1	2	3	4	5	
subitem not at all important	○	○	○	○	◉	essential

Does your paper address subitem 1b-iii?

Copy and paste relevant sections from the manuscript abstract (include quotes in quotation marks "like this" to indicate direct quotes from your manuscript), or elaborate on this item by providing additional information not in the ms, or briefly explain why the item is not applicable/relevant for your study

recruitment: "Young adults (age, 18-29 years) were recruited within 5 years of completing active cancer treatment by using the
Vivibot chatbot on Facebook messenger." Web-based assessments: "All participants
were assessed for psychosocial well-being via online surveys"

1b-iv) RESULTS section in abstract must contain use data

Report number of participants enrolled/assessed in each group, the use/uptake of the intervention (e.g., attrition/adherence metrics, use over time, number of logins etc.), in addition to primary/secondary outcomes. (Note: Only report in the abstract what the main paper is reporting. If this information is missing from the main body of text, consider adding it)

	1	2	3	4	5	
subitem not at all important	○	○	○	○	◉	essential

Does your paper address subitem 1b-iv?

Copy and paste relevant sections from the manuscript abstract (include quotes in quotation marks "like this" to indicate direct quotes from your manuscript), or elaborate on this item by providing additional information not in the ms, or briefly explain why the item is not applicable/relevant for your study

Data from 45 young adults (36 women; mean age: 25 [SD 2.9]; experimental group: n=25; control group: n=20) were
analyzed. Participants in the experimental group spent an average of 74 minutes across an average of 12 active sessions chatting
with Vivibot

1b-v) CONCLUSIONS/DISCUSSION in abstract for negative trials

Conclusions/Discussions in abstract for negative trials: Discuss the primary outcome - if the trial is negative (primary outcome not changed), and the intervention was not used, discuss whether negative results are attributable to lack of uptake and discuss reasons. (Note: Only report in the abstract what the main paper is reporting. If this information is missing from the main body of text, consider adding it)

	1	2	3	4	5	
subitem not at all important	○	○	○	○	◉	essential

Does your paper address subitem 1b-v?

Copy and paste relevant sections from the manuscript abstract (include quotes in quotation marks "like this" to indicate direct quotes from your manuscript), or elaborate on this item by providing additional information not in the ms, or briefly explain why the item is not applicable/relevant for your study

The chatbot format provides a useful and acceptable way of delivering positive psychology skills to young adults
who have undergone cancer treatment and supports anxiety reduction. Further analysis with a larger sample size is required to
confirm this consistent pattern.

INTRODUCTION

2a) In INTRODUCTION: Scientific background and explanation of rationale

2a-i) Problem and the type of system/solution

Describe the problem and the type of system/solution that is object of the study: intended as stand-alone intervention vs. incorporated in broader health care program? Intended for a particular patient population? Goals of the intervention, e.g., being more cost-effective to other interventions, replace or complement other solutions? (Note: Details about the intervention are provided in “Methods” under 5)

	1	2	3	4	5	
subitem not at all important	○	○	○	○	◉	essential

Does your paper address subitem 2a-i? *

Copy and paste relevant sections from the manuscript (include quotes in quotation marks "like this" to indicate direct quotes from your manuscript), or elaborate on this item by providing additional information not in the ms, or briefly explain why the item is not applicable/relevant for your study

The introduction details the heightened risk of mental health problems among young people after cancer treatment (population); the use of positive psychology skills to improve emotion among people diagnosed with serious illness (type of

intervention); and the promise of chatbots to deliver intervention to young people (type of intervention).

2a-ii) Scientific background, rationale: What is known about the (type of) system

Scientific background, rationale: What is known about the (type of) system that is the object of the study (be sure to discuss the use of similar systems for other conditions/diagnoses, if appropiate), motivation for the study, i.e. what are the reasons for and what is the context for this specific study, from which stakeholder viewpoint is the study performed, potential impact of findings [2]. Briefly justify the choice of the comparator.

	1	2	3	4	5	
subitem not at all important	○	○	○	○	◉	essential

Does your paper address subitem 2a-ii? *

Copy and paste relevant sections from the manuscript (include quotes in quotation marks "like this" to indicate direct quotes from your manuscript), or elaborate on this item by providing additional information not in the ms, or briefly explain why the item is not applicable/relevant for your study

We thoroughly review the literature on the use of positive psychology interventions to improve affect among people diagnosed with serious illness, the experiences of young people after cancer treatment, and the reasons we piloted a chatbot on social media for this population.

2b) In INTRODUCTION: Specific objectives or hypotheses

Does your paper address CONSORT subitem 2b? *

Copy and paste relevant sections from the manuscript (include quotes in quotation marks "like this" to indicate direct quotes from your manuscript), or elaborate on this item by providing additional information not in the ms, or briefly explain why the item is not applicable/relevant for your study

"The purpose of this
feasibility study was to evaluate engagement and usability of
the Vivibot chatbot. An additional goal was to evaluate the
preliminary effects of positive psychology skills delivered
through Vivibot on key psychosocial well-being outcomes in
young adults treated for cancer." "Our hypotheses were as follows: (1) After 4
weeks, exposure to Vivibot would result in decreased depression, anxiety, and
negative emotions and increased positive emotions compared
to the control condition. (2) Among the treatment group, greater
engagement in chatbot lessons would be associated with better
outcomes.

METHODS

3a) Description of trial design (such as parallel, factorial) including allocation ratio

Does your paper address CONSORT subitem 3a? *

Copy and paste relevant sections from the manuscript (include quotes in quotation marks "like this" to indicate direct quotes from your manuscript), or elaborate on this item by providing additional information not in the ms, or briefly explain why the item is not applicable/relevant for your study

"This was a 4-week pilot randomized controlled trial
evaluating feasibility, usability, and initial efficacy of the Vivibot
chatbot. At 4 weeks, control participants were given full access
to the 4 weeks of content in the experimental condition. An
8-week follow-up survey thus allowed for validation of the main
outcome analyses in the control group."

3b) Important changes to methods after trial commencement (such as eligibility criteria), with reasons

Does your paper address CONSORT subitem 3b? *

Copy and paste relevant sections from the manuscript (include quotes in quotation marks "like this" to indicate direct quotes from your manuscript), or elaborate on this item by providing additional information not in the ms, or briefly explain why the item is not applicable/relevant for your study

Results: "After excluding 6 control participants who were erroneously given access to chatbot content at 2 weeks instead of 4 weeks, the final analytic sample comprised 45 participants."

3b-i) Bug fixes, Downtimes, Content Changes

Bug fixes, Downtimes, Content Changes: ehealth systems are often dynamic systems. A description of changes to methods therefore also includes important changes made on the intervention or comparator during the trial (e.g., major bug fixes or changes in the functionality or content) (5-iii) and other “unexpected events” that may have influenced study design such as staff changes, system failures/downtimes, etc. [2].

	1	2	3	4	5	
subitem not at all important	(●)	○	○	○	○	essential

Does your paper address subitem 3b-i?

Copy and paste relevant sections from the manuscript (include quotes in quotation marks "like this" to indicate direct quotes from your manuscript), or elaborate on this item by providing additional information not in the ms, or briefly explain why the item is not applicable/relevant for your study

n/a no content changes were made during the trial

4a) Eligibility criteria for participants

Does your paper address CONSORT subitem 4a? *

Copy and paste relevant sections from the manuscript (include quotes in quotation marks "like this" to indicate direct quotes from your manuscript), or elaborate on this item by providing additional information not in the ms, or briefly explain why the item is not applicable/relevant for your study

"Participants were young adults, aged 18-29 years, consistent with the developmental literature [36] and the field of oncology [4]; English literate; and reported having a cancer diagnosis and completing treatment for cancer within 5 years of starting the study. They also had to have access to Facebook Messenger for the study duration (either through a Facebook or Instagram account). Participants were excluded if they did not meet age or cancer diagnosis or treatment requirements or were unable to access Vivibot through Facebook Messenger. They were not excluded based on the type of cancer diagnosis."

4a-i) Computer / Internet literacy

Computer / Internet literacy is often an implicit “de facto” eligibility criterion - this should be explicitly clarified.

	1	2	3	4	5	
subitem not at all important	○	○	○	○	◉	essential

Does your paper address subitem 4a-i?

Copy and paste relevant sections from the manuscript (include quotes in quotation marks "like this" to indicate direct quotes from your manuscript), or elaborate on this item by providing additional information not in the ms, or briefly explain why the item is not applicable/relevant for your study

" They also had to have access to Facebook Messenger for the study duration (either through a Facebook or Instagram account). "

4a-ii) Open vs. closed, web-based vs. face-to-face assessments:

Open vs. closed, web-based vs. face-to-face assessments: Mention how participants were recruited (online vs. offline), e.g., from an open access website or from a clinic, and clarify if this was a purely web-based trial, or there were face-to-face components (as part of the intervention or for assessment), i.e., to what degree got the study team to know the participant. In online-only trials, clarify if participants were quasi-anonymous and whether having multiple identities was possible or whether technical or logistical measures (e.g., cookies, email confirmation, phone calls) were used to detect/prevent these.

	1	2	3	4	5	
subitem not at all important	○	○	○	○	◉	essential

Does your paper address subitem 4a-ii? *

Copy and paste relevant sections from the manuscript (include quotes in quotation marks "like this" to indicate direct quotes from your manuscript), or elaborate on this item by providing additional information not in the ms, or briefly explain why the item is not applicable/relevant for your study

Online assessments through Qualtrics software (Salt Lake City, UT) were administered at baseline and weeks 2, 4, and 8.

4a-iii) Information giving during recruitment

Information given during recruitment. Specify how participants were briefed for recruitment and in the informed consent procedures (e.g., publish the informed consent documentation as appendix, see also item X26), as this information may have an effect on user self-selection, user expectation and may also bias results.

	1	2	3	4	5	
subitem not at all important	○	○	◉	○	○	essential

Does your paper address subitem 4a-iii?

Copy and paste relevant sections from the manuscript (include quotes in quotation marks "like this" to indicate direct quotes from your manuscript), or elaborate on this item by providing additional information not in the ms, or briefly explain why the item is not applicable/relevant for your study

The chatbot then randomized users 1:1 to one of two groups:
(1) immediate access to the full Vivibot chatbot content
(experimental group) or (2) access to daily emotion ratings

through Facebook Messenger with delayed full access to the full Vivibot chatbot only after 4 weeks (control group).

4b) Settings and locations where the data were collected

Does your paper address CONSORT subitem 4b? *

Copy and paste relevant sections from the manuscript (include quotes in quotation marks "like this" to indicate direct quotes from your manuscript), or elaborate on this item by providing additional information not in the ms, or briefly explain why the item is not applicable/relevant for your study

Introduction: "Hopelab.....created a chatbot called Vivibot,
delivered over Facebook messenger," Methods: "Participants were excluded if they did not meet age
or cancer diagnosis or treatment requirements or were unable
to access Vivibot through Facebook Messenger." "Enrollment was managed entirely
through the Vivibot chatbot." "Online assessments through Qualtrics software"

4b-i) Report if outcomes were (self-)assessed through online questionnaires

Clearly report if outcomes were (self-)assessed through online questionnaires (as common in web-based trials) or otherwise.

	1	2	3	4	5	
subitem not at all important	○	○	○	○	●	essential

Does your paper address subitem 4b-i? *

Copy and paste relevant sections from the manuscript (include quotes in quotation marks "like this" to indicate direct quotes from your manuscript), or elaborate on this item by providing additional information not in the ms, or briefly explain why the item is not applicable/relevant for your study

"Online assessments through Qualtrics software (Salt Lake City,
UT) were administered at baseline and weeks 2, 4, and 8"

4b-ii) Report how institutional affiliations are displayed

Report how institutional affiliations are displayed to potential participants [on ehealth media], as affiliations with prestigious hospitals or universities may affect volunteer rates, use, and reactions with regards to an intervention.(Not a required item – describe only if this may bias results)

	1	2	3	4	5	
subitem not at all important	◉	○	○	○	○	essential

Does your paper address subitem 4b-ii?

Copy and paste relevant sections from the manuscript (include quotes in quotation marks "like this" to indicate direct quotes from your manuscript), or elaborate on this item by providing additional information not in the ms, or briefly explain why the item is not applicable/relevant for your study

n/a, this would not bias results.

5) The interventions for each group with sufficient details to allow replication, including how and when they were actually administered

5-i) Mention names, credential, affiliations of the developers, sponsors, and owners

Mention names, credential, affiliations of the developers, sponsors, and owners [6] (if authors/evaluators are owners or developer of the software, this needs to be declared in a “Conflict of interest” section or mentioned elsewhere in the manuscript).

	1	2	3	4	5	
subitem not at all important	○	○	○	○	◉	essential

Does your paper address subitem 5-i?

Copy and paste relevant sections from the manuscript (include quotes in quotation marks "like this" to indicate direct quotes from your manuscript), or elaborate on this item by providing additional information not in the ms, or briefly explain why the item is not applicable/relevant for your study

The conflict of interest section of the manuscript details the authors' affiliation

with and relationship to Hopelab, the creator of the chatbot.

5-ii) Describe the history/development process

Describe the history/development process of the application and previous formative evaluations (e.g., focus groups, usability testing), as these will have an impact on adoption/use rates and help with interpreting results.

	1	2	3	4	5	
subitem not at all important	○	○	○	○	◉	essential

Does your paper address subitem 5-ii?

Copy and paste relevant sections from the manuscript (include quotes in quotation marks "like this" to indicate direct quotes from your manuscript), or elaborate on this item by providing additional information not in the ms, or briefly explain why the item is not applicable/relevant for your study

Before launching the pilot trial, Hopelab conducted formative work through interviews and focus groups with adolescents and young adults treated for cancer to refine content for the chatbot format and inform adaptation for delivery to a young userbase with a shared experience of cancer treatment. Upon completion of the focus groups, text was tailored based on their specific suggestions, and video and other content produced by young adults who were treated for cancer were incorporated directly into the chatbot. Vivibot additionally incorporated six daily emotion ratings (described in the Measures section) and periodic check-ins on participants' satisfaction with their interactions with the chatbot. The content is outlined in Multimedia Appendix 1, and sample user experience content is in Multimedia Appendix 2.

5-iii) Revisions and updating

Revisions and updating. Clearly mention the date and/or version number of the application/intervention (and comparator, if applicable) evaluated, or describe whether the intervention underwent major changes during the evaluation process, or whether the development and/or content was "frozen" during the trial. Describe dynamic components such as news feeds or changing content which may have an impact on the replicability of the intervention (for unexpected events see item 3b).

1 2 3 4 5

subitem not at all important	(●)	○	○	○	○	essential

Does your paper address subitem 5-iii?

Copy and paste relevant sections from the manuscript (include quotes in quotation marks "like this" to indicate direct quotes from your manuscript), or elaborate on this item by providing additional information not in the ms, or briefly explain why the item is not applicable/relevant for your study

n/a there were no revisions or updates to report.

5-iv) Quality assurance methods

Provide information on quality assurance methods to ensure accuracy and quality of information provided [1], if applicable.

	1	2	3	4	5	
subitem not at all important	○	○	○	○	(●)	essential

Does your paper address subitem 5-iv?

Copy and paste relevant sections from the manuscript (include quotes in quotation marks "like this" to indicate direct quotes from your manuscript), or elaborate on this item by providing additional information not in the ms, or briefly explain why the item is not applicable/relevant for your study

"Vivibot delivers a cognitive and behavioral intervention to increase positive emotion developed by Moskowitz et al (eg, [24]). The intervention was originally based on the Stress and Coping theory and the Broaden-and-Build theory of positive emotion and focused on the teaching and practice of eight positive psychological skills: noticing and acknowledging positive events, savoring positive events, gratitude, positive reappraisal, acts of kindness, mindfulness, personal strengths, and attainable goals. Rationale for inclusion of each of the positive emotion skills is provided elsewhere [38]. This core intervention was adapted for the chatbot format by creating seven conversational teaching lessons and seven practice lessons that were repeated three times to create 28 days of content. The eight skills were covered in seven lessons by combining acknowledging and savoring positive events into one lesson set."

one of the authors of the manuscript (Dr. Moskowitz) is the creator of the intervention on which Vivibot was based. In addition to all authors, she reviewed all content of Vivibot and the manuscript.

5-v) Ensure replicability by publishing the source code, and/or providing screenshots/screen-capture video, and/or providing flowcharts of the algorithms used

Ensure replicability by publishing the source code, and/or providing screenshots/screen-capture video, and/or providing flowcharts of the algorithms used. Replicability (i.e., other researchers should in principle be able to replicate the study) is a hallmark of scientific reporting.

	1	2	3	4	5	
subitem not at all important	○	○	○	○	◉	essential

Does your paper address subitem 5-v?

Copy and paste relevant sections from the manuscript (include quotes in quotation marks "like this" to indicate direct quotes from your manuscript), or elaborate on this item by providing additional information not in the ms, or briefly explain why the item is not applicable/relevant for your study

"The content is outlined in Multimedia
Appendix 1, and sample user experience content is in
Multimedia Appendix 2."

5-vi) Digital preservation

Digital preservation: Provide the URL of the application, but as the intervention is likely to change or disappear over the course of the years; also make sure the intervention is archived (Internet Archive, webcitation.org, and/or publishing the source code or screenshots/videos alongside the article). As pages behind login screens cannot be archived, consider creating demo pages which are accessible without login.

	1	2	3	4	5	
subitem not at all important	○	○	○	○	○	essential

Does your paper address subitem 5-vi?

Copy and paste relevant sections from the manuscript (include quotes in quotation marks "like this" to indicate direct quotes from your manuscript), or elaborate on this item by providing additional information not in the ms, or briefly explain why the item is not applicable/relevant for your study

Please see response to 5-v, webcitation is no longer taking submissions

Please see response to 5-v. Webcitation is no longer taking submissions.

5-vii) Access

Access: Describe how participants accessed the application, in what setting/context, if they had to pay (or were paid) or not, whether they had to be a member of specific group. If known, describe how participants obtained "access to the platform and Internet" [1]. To ensure access for editors/reviewers/readers, consider to provide a "backdoor" login account or demo mode for reviewers/readers to explore the application (also important for archiving purposes, see vi).

	1	2	3	4	5	
subitem not at all important	○	○	○	○	◉	essential

Does your paper address subitem 5-vii? *

Copy and paste relevant sections from the manuscript (include quotes in quotation marks "like this" to indicate direct quotes from your manuscript), or elaborate on this item by providing additional information not in the ms, or briefly explain why the item is not applicable/relevant for your study

Introduction: "Hopelab (San Francisco, CA) created a chatbot called Vivibot, delivered over Facebook messenger"; Methods: "Online assessments through Qualtrics software (Salt Lake City,
UT) were administered at baseline and weeks 2, 4, and 8.
Participants received US $20 Amazon gift cards for completing
each survey (a total of US $80 possible compensation)."

5-viii) Mode of delivery, features/functionalities/components of the intervention and comparator, and the theoretical framework

Describe mode of delivery, features/functionalities/components of the intervention and comparator, and the theoretical framework [6] used to design them (instructional strategy [1], behaviour change techniques, persuasive features, etc., see e.g., [7, 8] for terminology). This includes an in-depth description of the content (including where it is coming from and who developed it) [1]," whether [and how] it is tailored to individual circumstances and allows users to track their progress and receive feedback" [6]. This also includes a description of communication delivery channels and – if computer-mediated communication is a component – whether communication was synchronous or asynchronous [6]. It also includes information on presentation strategies [1], including page design principles, average amount of text on pages, presence of hyperlinks to other resources, etc. [1].

	1	2	3	4	5	
subitem not at all important	○	○	○	○	◉	essential

important

Does your paper address subitem 5-viii? *

Copy and paste relevant sections from the manuscript (include quotes in quotation marks "like this" to indicate direct quotes from your manuscript), or elaborate on this item by providing additional information not in the ms, or briefly explain why the item is not applicable/relevant for your study

"Vivibot
Vivibot is a chatbot designed to deliver prewritten and automatically delivered material to users online via a decision tree structure. Before study enrollment, users were explicitly told that they are chatting with an automated system (not a person) and periodically reminded of this throughout study participation.
Vivibot delivers a cognitive and behavioral intervention to increase positive emotion developed by Moskowitz et al (eg, [24]). The intervention was originally based on the Stress and Coping theory and the Broaden-and-Build theory of positive emotion and focused on the teaching and practice of eight positive psychological skills: noticing and acknowledging positive events, savoring positive events, gratitude, positive reappraisal, acts of kindness, mindfulness, personal strengths, and attainable goals. Rationale for inclusion of each of the positive emotion skills is provided elsewhere [38]. This core intervention was adapted for the chatbot format by creating seven conversational teaching lessons and seven practice lessons that were repeated three times to create 28 days of content. The eight skills were covered in seven lessons by combining acknowledging and savoring positive events into one lesson set.
Before launching the pilot trial, Hopelab conducted formative work through interviews and focus groups with adolescents and young adults treated for cancer to refine content for the chatbot format and inform adaptation for delivery to a young userbase with a shared experience of cancer treatment. Upon completion of the focus groups, text was tailored based on their specific suggestions, and video and other content produced by young adults who were treated for cancer were incorporated directly into the chatbot. Vivibot additionally incorporated six daily [illegible]

emotion ratings (described in the Measures section) and periodic check-ins on participants' satisfaction with their interactions with the chatbot. The content is outlined in Multimedia Appendix 1, and sample user experience content is in Multimedia Appendix 2.
Control
Control participants received delayed access to the full Vivibot chatbot. Those randomized to this group were given a message within the chatbot saying that their access to the full content would be delayed by 4 weeks. During this time, they were asked to report daily emotion ratings but received no other chatbot content. A set of six participants in the control condition encountered a technical error after the 2-week survey. This resulted in access to the full chatbot content at 2 weeks instead of the full 4 weeks. These six participants have been excluded from reported analyses to test outcomes at 4 weeks across a clean sample."

5-ix) Describe use parameters

Describe use parameters (e.g., intended “doses” and optimal timing for use). Clarify what instructions or recommendations were given to the user, e.g., regarding timing, frequency, heaviness of use, if any, or was the intervention used ad libitum.

	1	2	3	4	5	
subitem not at all important	(●)	( )	( )	( )	( )	essential

Does your paper address subitem 5-ix?

Copy and paste relevant sections from the manuscript (include quotes in quotation marks "like this" to indicate direct quotes from your manuscript), or elaborate on this item by providing additional information not in the ms, or briefly explain why the item is not applicable/relevant for your study

n/a, ad libitum

5-x) Clarify the level of human involvement

Clarify the level of human involvement (care providers or health professionals, also technical assistance) in the e-intervention or as co-intervention (detail number and expertise of professionals involved, if any, as well as “type of assistance offered, the timing and frequency of the support, how it is initiated, and the medium by which the assistance is delivered”. It may be necessary to distinguish between the level of human involvement required for the trial, and the level of human involvement required for a routine application outside of a RCT setting (discuss under item 21 – generalizability).

1 2 3 4 5

	1	2	3	4	5	
subitem not at all important	◉	○	○	○	○	essential

Does your paper address subitem 5-x?

Copy and paste relevant sections from the manuscript (include quotes in quotation marks "like this" to indicate direct quotes from your manuscript), or elaborate on this item by providing additional information not in the ms, or briefly explain why the item is not applicable/relevant for your study

n/a the intervention had no human involement

5-xi) Report any prompts/reminders used

Report any prompts/reminders used: Clarify if there were prompts (letters, emails, phone calls, SMS) to use the application, what triggered them, frequency etc. It may be necessary to distinguish between the level of prompts/reminders required for the trial, and the level of prompts/reminders for a routine application outside of a RCT setting (discuss under item 21 – generalizability).

	1	2	3	4	5	
subitem not at all important	○	○	◉	○	○	essential

Does your paper address subitem 5-xi? *

Copy and paste relevant sections from the manuscript (include quotes in quotation marks "like this" to indicate direct quotes from your manuscript), or elaborate on this item by providing additional information not in the ms, or briefly explain why the item is not applicable/relevant for your study

"Additionally, daily prompts for
emotion ratings were triggered in the chatbot in both the
experimental and control conditions. "

5-xii) Describe any co-interventions (incl. training/support)

Describe any co-interventions (incl. training/support): Clearly state any interventions that are provided in addition to the targeted eHealth intervention, as ehealth intervention may not be designed as stand-alone intervention. This includes training sessions and support [1]. It may be necessary to distinguish between the level of training required for the trial, and the level of training for a routine application outside of a RCT setting (discuss under item 21 – generalizability.

	1	2	3	4	5	
subitem not at all important	◉	○	○	○	○	essential

Does your paper address subitem 5-xii? *

Copy and paste relevant sections from the manuscript (include quotes in quotation marks "like this" to indicate direct quotes from your manuscript), or elaborate on this item by providing additional information not in the ms, or briefly explain why the item is not applicable/relevant for your study

n/a, no co-interventions

6a) Completely defined pre-specified primary and secondary outcome measures, including how and when they were assessed

Does your paper address CONSORT subitem 6a? *

Copy and paste relevant sections from the manuscript (include quotes in quotation marks "like this" to indicate direct quotes from your manuscript), or elaborate on this item by providing additional information not in the ms, or briefly explain why the item is not applicable/relevant for your study

"Measures
Chatbot Feasibility/Acceptability
Engagement With the Chatbot
Full conversational history with the chatbot was examined by session. An interaction was considered a session if there was engagement with the bot lasting at least two user inputs within 5 minutes and a break no longer than 5 minutes. The total number of sessions was calculated as the count of all sessions for an individual user, and the total interaction time with the chatbot was defined as the total time from the start to the end of a session summed across all sessions. In addition, an engaged session was defined as any session that included, at minimum, a completion of the 6-item emotion rating. Identifying engaged sessions was important because some interactions deemed sessions included only a participant receiving a notification to check-in and then responding that they were not available to talk. These sessions therefore did not include meaningful interactions with the chatbot content. Defining engaged sessions as requiring a user to progress to daily emotion rating completion allowed for comparisons of engaged sessions in both experimental and control groups.
Chatbot Feedback
At the completion of each skill in the experimental group, users were asked to rate how helpful they found the lesson of the day on a scale from 0 (“not really”) to 3 (“yes, very”). Mean ratings across all lessons were calculated for each participant (a given participant could have more than one rating). Participants in the experimental group were also given periodic opportunities to provide open-ended feedback about the chatbot program. On the seventh interaction, participants were specifically asked “How likely would you be to recommend Vivibot to a friend?” [illegible]

(rated on a scale from 0 to 10) and "Why did you give that score." These questions were used to assess how much participants enjoyed the chatbot and what they found particularly valuable or not valuable.

Well-Being Outcomes

Anxiety and Depression Symptoms

Anxiety and depression symptoms were measured by assessments from the Patient-Reported Outcomes Measurement Information System (PROMIS) initiative [39]. PROMIS measures are listed as "emerging measures" for further research by the American Psychiatric Association and published in the Diagnostic and Statistical Manual of Mental Disorders - fifth edition [40]. Anxiety symptoms were measured by the 4-item PROMIS Emotional Distress-Anxiety, Short Form [39]. This measure has demonstrated clinical validity in patients with chronic health conditions including cancer [41] as well as pediatric patients [42]. Depression symptoms were measured by the 4-item PROMIS Emotional Distress-Depression, Short Form [39], which captures self-reported depression symptoms, and performs similar to legacy measures (Beck Depression Inventory, Center for Epidemiological Studies Depression) in adults diagnosed with cancer [43]. Both PROMIS measures asked participants to indicate the frequency of symptoms over the past 7 days on a five-point response scale (0-4). Item scores were summed to obtain the total raw score, which was then converted to a T score (mean 50, SD 10). The ranges are as follows: anxiety: t =40.3-81.6 and depression: t =41.0-79.4. The cutoffs on both scales are as follows: mild, t =55; moderate, t =60; and severe, t =70 [44].

Positive and Negative Emotions

Two-week retrospective reports of positive and negative emotions were measured with the modified version of the Differential Emotions Scale, which includes additional items to measure positive emotion [45]. Positive emotions included 10 items scored from 0 ("not at all") to 4 ("most of the time"), with ranges from 0 to 40. Negative emotions included 9 items scored similarly (range: 0-36). Additionally, daily prompts for emotion ratings were triggered in the chatbot in both the experimental and control conditions. Six discrete emotions (happy, excited, content, worried, irritable/angry, and sad) were rated on a scale from 0 ("not at all") to 8 ("extremely").

Multimedia Appendix 3 presents a depiction of this measure.
Responses from the three positive and three negative emotion items were each averaged to make a single positive and negative emotion score (range: 0-24) for each time period of interest (daily for daily prompts and at each 2- and 4-week surveys)."

6a-i) Online questionnaires: describe if they were validated for online use and apply CHERRIES items to describe how the questionnaires were designed/deployed

If outcomes were obtained through online questionnaires, describe if they were validated for online use and apply CHERRIES items to describe how the questionnaires were designed/deployed [9].

	1	2	3	4	5	
subitem not at all important	◉	○	○	○	○	essential

Does your paper address subitem 6a-i?

Copy and paste relevant sections from manuscript text

n/a all validation data were reported as above and no formal validation of online items was conducted for this study

6a-ii) Describe whether and how “use” (including intensity of use/dosage) was defined/measured/monitored

Describe whether and how “use” (including intensity of use/dosage) was defined/measured/monitored (logins, logfile analysis, etc.). Use/adoption metrics are important process outcomes that should be reported in any ehealth trial.

	1	2	3	4	5	
subitem not at all important	○	○	○	○	◉	essential

Does your paper address subitem 6a-ii?

Copy and paste relevant sections from manuscript text

"Measures
Chatbot Feasibility/Acceptability
Engagement With the Chatbot
Full conversational history with the chatbot was examined by session. An interaction was considered a session if there was engagement with the bot lasting at least two user inputs within 5 minutes and a break no longer than 5 minutes. The total number of sessions was calculated as the count of all sessions for an individual user, and the total interaction time with the chatbot was defined as the total time from the start to the end of a session summed across all sessions. In addition, an engaged session was defined as any session that included, at minimum, a completion of the 6-item emotion rating. Identifying engaged sessions was important because some interactions deemed sessions included only a participant receiving a notification to check-in and then responding that they were not available to talk. These sessions therefore did not include meaningful interactions with the chatbot content. Defining engaged sessions as requiring a user to progress to daily emotion rating completion allowed for comparisons of engaged sessions in both experimental and control groups."

6a-iii) Describe whether, how, and when qualitative feedback from participants was obtained

Describe whether, how, and when qualitative feedback from participants was obtained (e.g., through emails, feedback forms, interviews, focus groups).

	1	2	3	4	5	
subitem not at all important	○	○	○	○	◉	essential

Does your paper address subitem 6a-iii?

Copy and paste relevant sections from manuscript text

"Chatbot Feedback
At the completion of each skill in the experimental group, users were asked to rate how helpful they found the lesson of the day on a scale from 0 (“not really”) to 3 (“yes, very”). Mean ratings across all lessons were calculated for each participant (a given participant could have more than one rating). Participants in the experimental group were also given periodic opportunities to provide open-ended feedback about the chatbot program. On the seventh interaction, participants were specifically asked “How likely would you be to recommend Vivibot to a friend?” (rated on a scale from 0 to 10) and “Why did you give that score.” These questions were used to assess how much participants enjoyed the chatbot and what they found particularly valuable or not valuable."

6b) Any changes to trial outcomes after the trial commenced, with reasons

Does your paper address CONSORT subitem 6b? *

Copy and paste relevant sections from the manuscript (include quotes in quotation marks "like this" to indicate direct quotes from your manuscript), or elaborate on this item by providing additional information not in the ms, or briefly explain why the item is not applicable/relevant for your study

N/A, no changes were made

7a) How sample size was determined

NPT: When applicable, details of whether and how the clustering by care provides or centers was addressed

7a-i) Describe whether and how expected attrition was taken into account when calculating the sample size

Describe whether and how expected attrition was taken into account when calculating the sample size.

	1	2	3	4	5	
subitem not at all important	(●)	( )	( )	( )	( )	essential

Does your paper address subitem 7a-i?

Copy and paste relevant sections from manuscript title (include quotes in quotation marks "like this" to indicate direct quotes from your manuscript), or elaborate on this item by providing additional information not in the ms, or briefly explain why the item is not applicable/relevant for your study

this was a small pilot feasibility trial not powered to detect any effects on health outcomes.

7b) When applicable, explanation of any interim analyses and stopping guidelines

Does your paper address CONSORT subitem 7b? *

Copy and paste relevant sections from the manuscript (include quotes in quotation marks "like this" to indicate direct quotes from your manuscript), or elaborate on this item by providing additional information not in the ms, or briefly explain why the item is not applicable/relevant for your study

n/a

8a) Method used to generate the random allocation sequence

NPT: When applicable, how care providers were allocated to each trial group

Does your paper address CONSORT subitem 8a? *

Copy and paste relevant sections from the manuscript (include quotes in quotation marks "like this" to indicate direct quotes from your manuscript), or elaborate on this item by providing additional

to indicate direct quotes from your manuscript), or elaborate on this item by providing additional information not in the ms, or briefly explain why the item is not applicable/relevant for your study

Methods: "The chatbot then randomized users 1:1 to one of two groups"

8b) Type of randomisation; details of any restriction (such as blocking and block size)

Does your paper address CONSORT subitem 8b? *

Copy and paste relevant sections from the manuscript (include quotes in quotation marks "like this" to indicate direct quotes from your manuscript), or elaborate on this item by providing additional information not in the ms, or briefly explain why the item is not applicable/relevant for your study

see above

9) Mechanism used to implement the random allocation sequence (such as sequentially numbered containers), describing any steps taken to conceal the sequence until interventions were assigned

Does your paper address CONSORT subitem 9? *

Copy and paste relevant sections from the manuscript (include quotes in quotation marks "like this" to indicate direct quotes from your manuscript), or elaborate on this item by providing additional information not in the ms, or briefly explain why the item is not applicable/relevant for your study

n/a

10) Who generated the random allocation sequence, who enrolled participants, and who assigned participants to interventions

Does your paper address CONSORT subitem 10? *

Copy and paste relevant sections from the manuscript (include quotes in quotation marks "like this" to indicate direct quotes from your manuscript), or elaborate on this item by providing additional information not in the ms, or briefly explain why the item is not applicable/relevant for your study

The chatbot was programmed to develop a random allocation sequence.

11a) If done, who was blinded after assignment to interventions (for example, participants, care providers, those assessing outcomes) and how

NPT: Whether or not administering co-interventions were blinded to group assignment

11a-i) Specify who was blinded, and who wasn't

Specify who was blinded, and who wasn't. Usually, in web-based trials it is not possible to blind the participants [1, 3] (this should be clearly acknowledged), but it may be possible to blind outcome assessors, those doing data analysis or those administering co-interventions (if any).

	1	2	3	4	5	
subitem not at all important	○	○	○	○	○	essential

Does your paper address subitem 11a-i? *

Copy and paste relevant sections from the manuscript (include quotes in quotation marks "like this" to indicate direct quotes from your manuscript), or elaborate on this item by providing additional information not in the ms, or briefly explain why the item is not applicable/relevant for your study

n/a this was an entirely digital trial - participants in each group were told the condition upon study enrollment, and there was no other human involvement during the trial, making researcher blinding not applicable.

11a-ii) Discuss e.g., whether participants knew which intervention was the "intervention of interest" and which one was the "comparator"

Informed consent procedures (4a-ii) can create biases and certain expectations - discuss e.g., whether participants knew which intervention was the "intervention of interest" and which one was the "comparator".

	1	2	3	4	5	
subitem not at all important	○	○	●	○	○	essential

Does your paper address subitem 11a-ii?

Copy and paste relevant sections from the manuscript (include quotes in quotation marks "like this" to indicate direct quotes from your manuscript), or elaborate on this item by providing additional information not in the ms, or briefly explain why the item is not applicable/relevant for your study

"The chatbot then randomized users 1:1 to one of two groups: (1) immediate access to the full Vivibot chatbot content (experimental group) or (2) access to daily emotion ratings through Facebook Messenger with delayed full access to the full Vivibot chatbot only after 4 weeks (control group)."

11b) If relevant, description of the similarity of interventions

(this item is usually not relevant for ehealth trials as it refers to similarity of a placebo or sham intervention to a active medication/intervention)

Does your paper address CONSORT subitem 11b? *

Copy and paste relevant sections from the manuscript (include quotes in quotation marks "like this" to indicate direct quotes from your manuscript), or elaborate on this item by providing additional information not in the ms, or briefly explain why the item is not applicable/relevant for your study

n/a

12a) Statistical methods used to compare groups for primary and secondary outcomes

NPT: When applicable, details of whether and how the clustering by care providers or centers was addressed

Does your paper address CONSORT subitem 12a? *

Copy and paste relevant sections from the manuscript (include quotes in quotation marks "like this" to indicate direct quotes from your manuscript), or elaborate on this item by providing additional information not in the ms, or briefly explain why the item is not applicable/relevant for your study

"Chatbot Engagement
Means and SDs were reported for time spent on all sessions. Within the experimental group, means and SDs for chatbot feedback were reported based on the measures described above. Open-ended feedback was evaluated, and quotes were selected to exemplify prominent themes.
Well-Being Outcomes
Well-being outcomes were assessed using a series of multilevel mixed-effects linear models for intention to treat. Multilevel models were used because they accommodate missing data and nonindependence in observations. For each construct (primary: anxiety, depression, positive/negative emotions; secondary: positive mental health symptoms, purpose, self-efficacy for coping with cancer, general self-efficacy, and loneliness), a separate mixed-effects model was used to evaluate the difference in magnitude of change from baseline to week 4 follow-up assessment as a function of intervention condition (experimental vs control) and the interaction of time by condition. Each model was evaluated on the basis of statistical significance (P<.05) of the interaction term. Given the pilot nature of the trial, in cases in which the P-value approached significance, effect sizes were also examined for strength and clinical meaningful differences in main outcomes across groups.
To validate results, we separately modeled changes in primary outcomes within the control condition only to examine changes in primary outcomes from the 4-week survey (when the control condition received access to the intervention) to the 8-week follow-up (which allowed for a possible 4 week intervention window, similar to that of the experimental group). This model did not include any condition or interaction term.
To examine the relationship between chatbot engagement and [illegible]

well-being outcomes, we used mixed-effects models to separately model each outcome as a function of the number of engaged sessions between baseline and follow-up, intervention condition (experimental vs control), and the interaction of engaged sessions and condition."

12a-i) Imputation techniques to deal with attrition / missing values

Imputation techniques to deal with attrition / missing values: Not all participants will use the intervention/comparator as intended and attrition is typically high in ehealth trials. Specify how participants who did not use the application or dropped out from the trial were treated in the statistical analysis (a complete case analysis is strongly discouraged, and simple imputation techniques such as LOCF may also be problematic [4]).

	1	2	3	4	5	
subitem not at all important	(•)	( )	( )	( )	( )	essential

Does your paper address subitem 12a-i? *

Copy and paste relevant sections from the manuscript (include quotes in quotation marks "like this" to indicate direct quotes from your manuscript), or elaborate on this item by providing additional information not in the ms, or briefly explain why the item is not applicable/relevant for your study

n/a no imputation was used

12b) Methods for additional analyses, such as subgroup analyses and adjusted analyses

Does your paper address CONSORT subitem 12b? *

Copy and paste relevant sections from the manuscript (include quotes in quotation marks "like this" to indicate direct quotes from your manuscript), or elaborate on this item by providing additional information not in the ms, or briefly explain why the item is not applicable/relevant for your study

n/a

X26) REB/IRB Approval and Ethical Considerations [recommended as subheading under "Methods"] (not a CONSORT item)

X26-i) Comment on ethics committee approval

	1	2	3	4	5	
subitem not at all important	○	○	○	○	◉	essential

Does your paper address subitem X26-i?

Copy and paste relevant sections from the manuscript (include quotes in quotation marks "like this" to indicate direct quotes from your manuscript), or elaborate on this item by providing additional information not in the ms, or briefly explain why the item is not applicable/relevant for your study

The Ethical and Independent Review Services [37] board approved all study procedures.

x26-ii) Outline informed consent procedures

Outline informed consent procedures e.g., if consent was obtained offline or online (how? Checkbox, etc.?), and what information was provided (see 4a-ii). See [6] for some items to be included in informed consent documents.

	1	2	3	4	5	
subitem not at all important	○	○	○	○	○	essential

Does your paper address subitem X26-ii?

Copy and paste relevant sections from the manuscript (include quotes in quotation marks "like this" to indicate direct quotes from your manuscript), or elaborate on this item by providing additional information not in the ms, or briefly explain why the item is not applicable/relevant for your study

"Those eligible and interested were sent an identification code and link to the study consent survey via Qualtrics."

X26-iii) Safety and security procedures

Safety and security procedures, incl. privacy considerations, and any steps taken to reduce the likelihood or detection of harm (e.g., education and training, availability of a hotline)

	1	2	3	4	5	
subitem not at all important	○	○	○	○	◉	essential

Does your paper address subitem X26-iii?

Copy and paste relevant sections from the manuscript (include quotes in quotation marks "like this" to indicate direct quotes from your manuscript), or elaborate on this item by providing additional information not in the ms, or briefly explain why the item is not applicable/relevant for your study

Vivibot included an automated assessment of risk, based on natural language processing, and delivered resources for crisis intervention if any of the crisis words were detected.

RESULTS

13a) For each group, the numbers of participants who were randomly assigned, received intended treatment, and were analysed for the primary outcome

NPT: The number of care providers or centers performing the intervention in each group and the number of patients treated by each care provider in each center

Does your paper address CONSORT subitem 13a? *

Copy and paste relevant sections from the manuscript (include quotes in quotation marks "like this" to indicate direct quotes from your manuscript), or elaborate on this item by providing additional information not in the ms, or briefly explain why the item is not applicable/relevant for your study

"In total, 51 participants completed a baseline assessment and were randomized to a study condition (experimental group: 25; control group: 26; Figure 1). After excluding 6 control participants who were erroneously given access to chatbot content at 2 weeks instead of 4 weeks, the final analytic sample comprised 45 participants."

13b) For each group, losses and exclusions after randomisation, together with reasons

Does your paper address CONSORT subitem 13b? (NOTE: Preferably, this is shown in a CONSORT flow diagram) *

Copy and paste relevant sections from the manuscript (include quotes in quotation marks "like this" to indicate direct quotes from your manuscript), or elaborate on this item by providing additional information not in the ms, or briefly explain why the item is not applicable/relevant for your study

Figure 1 (flow diagram) contains this information

13b-i) Attrition diagram

Strongly recommended: An attrition diagram (e.g., proportion of participants still logging in or using the intervention/comparator in each group plotted over time, similar to a survival curve) or other figures or tables demonstrating usage/dose/engagement.

	1	2	3	4	5	
subitem not at all important	◉	○	○	○	○	essential

Does your paper address subitem 13b-i?

Copy and paste relevant sections from the manuscript or cite the figure number if applicable (include quotes in quotation marks "like this" to indicate direct quotes from your manuscript), or elaborate on this item by providing additional information not in the ms, or briefly explain why the item is not applicable/relevant for your study

"The rate of follow-up survey
completion was 73% (33/45) at 2 weeks, 73% (33/45) at 4
weeks, and 58% (26/45) at 8 weeks.

14a) Dates defining the periods of recruitment and follow-up

Does your paper address CONSORT subitem 14a? *

Copy and paste relevant sections from the manuscript (include quotes in quotation marks "like this" to indicate direct quotes from your manuscript), or elaborate on this item by providing additional information not in the ms, or briefly explain why the item is not applicable/relevant for your study

recruitment took place over 3 months.

14a-i) Indicate if critical “secular events” fell into the study period

Indicate if critical “secular events” fell into the study period, e.g., significant changes in Internet resources available or “changes in computer hardware or Internet delivery resources”

	1	2	3	4	5	
subitem not at all important	(selected)	○	○	○	○	essential

Does your paper address subitem 14a-i?

Copy and paste relevant sections from the manuscript (include quotes in quotation marks "like this" to indicate direct quotes from your manuscript), or elaborate on this item by providing additional information not in the ms, or briefly explain why the item is not applicable/relevant for your study

n/a no events ocurred

14b) Why the trial ended or was stopped (early)

Does your paper address CONSORT subitem 14b? *

Copy and paste relevant sections from the manuscript (include quotes in quotation marks "like this" to indicate direct quotes from your manuscript), or elaborate on this item by providing additional information not in the ms, or briefly explain why the item is not applicable/relevant for your study

the trial was not stopped early

15) A table showing baseline demographic and clinical characteristics for each group

NPT: When applicable, a description of care providers (case volume, qualification, expertise, etc.) and centers (volume) in each group

Does your paper address CONSORT subitem 15? *

Copy and paste relevant sections from the manuscript (include quotes in quotation marks "like this" to indicate direct quotes from your manuscript), or elaborate on this item by providing additional information not in the ms, or briefly explain why the item is not applicable/relevant for your study

table 1

15-i) Report demographics associated with digital divide issues

In ehealth trials it is particularly important to report demographics associated with digital divide issues, such as age, education, gender, social-economic status, computer/Internet/ehealth literacy of the participants, if known.

	1	2	3	4	5	
subitem not at all important	○	○	○	○	◉	essential

Does your paper address subitem 15-i? *

Copy and paste relevant sections from the manuscript (include quotes in quotation marks "like this" to indicate direct quotes from your manuscript), or elaborate on this item by providing additional information not in the ms, or briefly explain why the item is not applicable/relevant for your study

table 1

16) For each group, number of participants (denominator) included in each analysis and whether the analysis was by original assigned groups

16-i) Report multiple “denominators” and provide definitions

Report multiple “denominators” and provide definitions: Report N’s (and effect sizes) “across a range of study participation [and use] thresholds” [1], e.g., N exposed, N consented, N used more than x times, N used more than y weeks, N participants “used” the intervention/comparator at specific pre-defined time points of interest (in absolute and relative numbers per group). Always clearly define “use” of the intervention.

	1	2	3	4	5	
subitem not at all important	○	○	○	○	○	essential

Does your paper address subitem 16-i? *

Copy and paste relevant sections from the manuscript (include quotes in quotation marks "like this" to indicate direct quotes from your manuscript), or elaborate on this item by providing additional information not in the ms, or briefly explain why the item is not applicable/relevant for your study

"Chatbot Engagement
During the 4 active weeks of the study, the experimental group spent an average of 73.8 (SD 52) min across an average of 12.1 (SD 7.1) engaged sessions chatting with Vivibot. The control group spent an average of 27.13 (SD 15.8min) across an average of 18.1 (SD 8.6) engaged sessions completing the 6-item emotion ratings only."

16-ii) Primary analysis should be intent-to-treat

Primary analysis should be intent-to-treat, secondary analyses could include comparing only “users”, with the appropriate caveats that this is no longer a randomized sample (see 18-i).

	1	2	3	4	5	
subitem not at all important	◉	○	○	○	○	essential

Does your paper address subitem 16-ii?

Copy and paste relevant sections from the manuscript (include quotes in quotation marks "like this" to indicate direct quotes from your manuscript), or elaborate on this item by providing additional information not in the ms, or briefly explain why the item is not applicable/relevant for your study

Primary analysis was engagement, depression and anxiety analyses used all available data.

17a) For each primary and secondary outcome, results for each group, and the estimated effect size and its precision (such as 95% confidence interval)

Does your paper address CONSORT subitem 17a? *

Copy and paste relevant sections from the manuscript (include quotes in quotation marks "like this" to indicate direct quotes from your manuscript), or elaborate on this item by providing additional information not in the ms, or briefly explain why the item is not applicable/relevant for your study

"Anxiety and Depression Symptoms
At baseline, both experimental and control groups presented with moderate levels of anxiety (experimental group: mean 64.5 [SD 6.1]; control group: mean 62.6 [SD 7.9]; threshold=60) and mild levels of depression (experimental group: mean 60.1 [SD 7.4]; control group: mean 59.0 [SD 9.2]; threshold=60).
Participants in the experimental group reported a greater reduction in anxiety than the control group at a trend level of statistical significance and small-to-moderate effect size (experimental reduction of 2.58 t-score units vs control of 0.7 units; z=–1.70; Cohen d=–0.41; P=.09; Table 2). Both experimental and control groups showed slight decreases in depressive symptom ratings with no evidence of a condition by time interaction (experimental reduction=1.83; control reduction=1.38; z=0.30; Cohen d=0.09; P=.77).
To validate the results, a post hoc analysis of the change between 4 weeks and 8 weeks in the control group (N=17), after receiving access to the full 28-day chatbot content, revealed a similar magnitude drop in anxiety symptoms (2.72 standardized points) and a statistical trend (P=.13). As in the experimental group, there was no significant reduction in depression in this subsample (Multimedia Appendix 4 presents for full results at 8 weeks).
Anxiety Symptoms by Engagement
When using engaged sessions to predict anxiety outcomes (as opposed to timepoint alone), there was a stronger trend-level relationship for group by engaged sessions interaction (z=–1.9, P=.06). There was no such trend for depression outcomes (z=–0.60, P=.55).
Positive and Negative Emotions
Both the experimental group and the control group showed a

similar magnitude decrease in negative emotion in retrospective emotion ratings from baseline to week 4 (mean difference: experimental group: –0.31, control group: –0.23), with no significant or trend-level interaction by group for negative emotion at 4 weeks (z=0.23; Cohen d=–0.01; P=.97). This pattern was also reflected in the daily emotion ratings recorded in the chatbot, which showed a significant decrease in negative emotion reporting (z=–2.44; P=.02), but no significant interaction between groups (z=–0.74; P=.46).
Both the experimental group and control group showed almost no change in retrospective positive emotion ratings from baseline to week 4 (mean difference: experimental group: 0.04, control group: –0.08). Interestingly, the daily emotion ratings reported directly in the chatbot showed a significant main effect of time for increased positive emotion across groups (z=3.56; P<.01). Further, there was a significant interaction by group (z=–2.07; P=.04); however, this effect was in the opposite direction from our hypothesis, as the control group showed greater increases in positive daily emotion ratings reported directly in the chatbot, compared to the experimental group."

17a-i) Presentation of process outcomes such as metrics of use and intensity of use

In addition to primary/secondary (clinical) outcomes, the presentation of process outcomes such as metrics of use and intensity of use (dose, exposure) and their operational definitions is critical. This does not only refer to metrics of attrition (13-b) (often a binary variable), but also to more continuous exposure metrics such as “average session length”. These must be accompanied by a technical description how a metric like a “session” is defined (e.g., timeout after idle time) [1] (report under item 6a).

	1	2	3	4	5	
subitem not at all important	○	○	○	○	◉	essential

Does your paper address subitem 17a-i?

Copy and paste relevant sections from the manuscript (include quotes in quotation marks "like this" to indicate direct quotes from your manuscript), or elaborate on this item by providing additional information not in the ms, or briefly explain why the item is not applicable/relevant for your study

"Chatbot Engagement
During the 4 active weeks of the study, the experimental group spent an average of 73.8 (SD 52) min across an average of 12.1 (SD 7.1) engaged sessions chatting with Vivibot. The control

[illegible] engaged sessions chatting with Vivibot. The control group spent an average of 27.13 (SD 15.8min) across an average of 18.1 (SD 8.6) engaged sessions completing the 6-item emotion ratings only."

17b) For binary outcomes, presentation of both absolute and relative effect sizes is recommended

Does your paper address CONSORT subitem 17b? *

Copy and paste relevant sections from the manuscript (include quotes in quotation marks "like this" to indicate direct quotes from your manuscript), or elaborate on this item by providing additional information not in the ms, or briefly explain why the item is not applicable/relevant for your study

n/a no binary outcomes

18) Results of any other analyses performed, including subgroup analyses and adjusted analyses, distinguishing pre-specified from exploratory

Does your paper address CONSORT subitem 18? *

Copy and paste relevant sections from the manuscript (include quotes in quotation marks "like this" to indicate direct quotes from your manuscript), or elaborate on this item by providing additional information not in the ms, or briefly explain why the item is not applicable/relevant for your study

n/a no subgroup analyses

18-i) Subgroup analysis of comparing only users

A subgroup analysis of comparing only users is not uncommon in ehealth trials, but if done, it must be stressed that this is a self-selected sample and no longer an unbiased sample from a randomized trial (see 16-iii).

	1	2	3	4	5	
subitem not at all important	(●)	( )	( )	( )	( )	essential

Does your paper address subitem 18-i?

Copy and paste relevant sections from the manuscript (include quotes in quotation marks "like this" to indicate direct quotes from your manuscript), or elaborate on this item by providing additional information not in the ms, or briefly explain why the item is not applicable/relevant for your study

n/a

19) All important harms or unintended effects in each group

(for specific guidance see CONSORT for harms)

Does your paper address CONSORT subitem 19? *

Copy and paste relevant sections from the manuscript (include quotes in quotation marks "like this" to indicate direct quotes from your manuscript), or elaborate on this item by providing additional information not in the ms, or briefly explain why the item is not applicable/relevant for your study

n/a no harms or unintended effects occurred.

19-i) Include privacy breaches, technical problems

Include privacy breaches, technical problems. This does not only include physical “harm” to participants, but also incidents such as perceived or real privacy breaches [1], technical problems, and other unexpected/unintended incidents. “Unintended effects” also includes unintended positive effects [2].

	1	2	3	4	5	
subitem not at all important	◉	○	○	○	○	essential

Does your paper address subitem 19-i?

Copy and paste relevant sections from the manuscript (include quotes in quotation marks "like this" to indicate direct quotes from your manuscript), or elaborate on this item by providing additional information not in the ms, or briefly explain why the item is not applicable/relevant for your study

n/a no privacy breeches ocurred

19-ii) Include qualitative feedback from participants or observations from staff/researchers

Include qualitative feedback from participants or observations from staff/researchers, if available, on strengths and shortcomings of the application, especially if they point to unintended/unexpected effects or uses. This includes (if available) reasons for why people did or did not use the application as intended by the developers.

	1	2	3	4	5
subitem not at all	[illegible]	[illegible]	[illegible]	[illegible]	[illegible]

subitem not at all important ○ ○ ○ ○ ◉ essential

Does your paper address subitem 19-ii?

Copy and paste relevant sections from the manuscript (include quotes in quotation marks "like this" to indicate direct quotes from your manuscript), or elaborate on this item by providing additional information not in the ms, or briefly explain why the item is not applicable/relevant for your study

"Perceived Helpfulness and Open-Ended Feedback.
On average, participants in the experimental group rated their experience of chatting with Vivibot as helpful, with an average rating of 2.03/3 (SD 0.72; range: 0-3). They were also likely to recommend Vivibot to a friend, with an average rating of 6.9/10 (SD 2.6; range: 0-10). When asked why they were likely or unlikely to recommend Vivibot to a friend, participants remarked on the utility and nonjudgmental nature of talking to an automated agent:
When going through treatment it was hard not to bum out my friends talking about treatments and life. My whole perspective changed. And this is a way to openly talk about those changes and you present great paths to take those thoughts rather than trying to internalize or face those awkward conversations with healthy friends.
Additional themes related to having a shared experience with others who had undergone cancer treatment and being able to just “vent”:
Because as weird as it is talking to a robot, it’s nice to vent and be able to see others with cancer talking and speaking out about how they coped or felt during their treatment. Seeing that I’m not alone and having someone guide me to find the positives in my life now is really helpful.
Participants also particularly enjoyed the positive psychology content itself:
You give me new perspectives on things and help me set goals for myself and find things to be thankful for.
I also like the lessons you share…like looking for the good in bad situations or setting the goals to do random acts of kindness.
Participants who gave lower ratings of Vivibot were less specific in their feedback:
I just haven’t found it very helpful.
This bot kind of makes me feel like I’m being talked at rather than talking with.
Vivbot is annoying.

Full quotes from participants can be found in Multimedia Appendix 5."

DISCUSSION

22) Interpretation consistent with results, balancing benefits and harms, and considering other relevant evidence

NPT: In addition, take into account the choice of the comparator, lack of or partial blinding, and unequal expertise of care providers or centers in each group

22-i) Restate study questions and summarize the answers suggested by the data, starting with primary outcomes and process outcomes (use)

Restate study questions and summarize the answers suggested by the data, starting with primary outcomes and process outcomes (use).

	1	2	3	4	5	
subitem not at all important	○	○	○	○	◉	essential

Does your paper address subitem 22-i? *

Copy and paste relevant sections from the manuscript (include quotes in quotation marks "like this" to indicate direct quotes from your manuscript), or elaborate on this item by providing additional information not in the ms, or briefly explain why the item is not applicable/relevant for your study

see Section "Principal Finidngs" of the discussion

22-ii) Highlight unanswered new questions, suggest future research

Highlight unanswered new questions, suggest future research.

	1	2	3	4	5	
subitem not at all important	○	○	○	○	◉	essential

Does your paper address subitem 22-ii?

Copy and paste relevant sections from the manuscript (include quotes in quotation marks "like this" to indicate direct quotes from your manuscript), or elaborate on this item by providing additional information not in the ms, or briefly explain why the item is not applicable/relevant for your study

" In
addition to a larger trial, future research should consider whether
the tool is as effective when delivered to other populations or
platforms."

20) Trial limitations, addressing sources of potential bias, imprecision, and, if relevant, multiplicity of analyses

20-i) Typical limitations in ehealth trials

Typical limitations in ehealth trials: Participants in ehealth trials are rarely blinded. Ehealth trials often look at a multiplicity of outcomes, increasing risk for a Type I error. Discuss biases due to non-use of the intervention/usability issues, biases through informed consent procedures, unexpected events.

	1	2	3	4	5	
subitem not at all important	○	○	○	○	◉	essential

Does your paper address subitem 20-i? *

Copy and paste relevant sections from the manuscript (include quotes in quotation marks "like this" to indicate direct quotes from your manuscript), or elaborate on this item by providing additional information not in the ms, or briefly explain why the item is not applicable/relevant for your study

See "Limitations" section of the discussion.

21) Generalisability (external validity, applicability) of the trial findings

NPT: External validity of the trial findings according to the intervention, comparators, patients, and care providers or centers involved in the trial

21-i) Generalizability to other populations

Generalizability to other populations: In particular, discuss generalizability to a general Internet population, outside of a RCT setting, and general patient population, including applicability of the study results for other organizations

	1	2	3	4	5	
subitem not at all important	◉	○	○	○	○	essential

Does your paper address subitem 21-i?

Copy and paste relevant sections from the manuscript (include quotes in quotation marks "like this" to indicate direct quotes from your manuscript), or elaborate on this item by providing additional information not in the ms, or briefly explain why the item is not applicable/relevant for your study

this intervention does not generalize to populations other than young adult cancer survivors.

21-ii) Discuss if there were elements in the RCT that would be different in a routine application setting

Discuss if there were elements in the RCT that would be different in a routine application setting (e.g., prompts/reminders, more human involvement, training sessions or other co-interventions) and what impact the omission of these elements could have on use, adoption, or outcomes if the intervention is applied outside of a RCT setting.

	1	2	3	4	5	
subitem not at all important	◉	○	○	○	○	essential

Does your paper address subitem 21-ii?

Copy and paste relevant sections from the manuscript (include quotes in quotation marks "like this" to indicate direct quotes from your manuscript), or elaborate on this item by providing additional information not in the ms, or briefly explain why the item is not applicable/relevant for your study

The trial was conducted entirely online, in the relatively real world environment of Facebook messenger. Participant payment for survey completion was different than a routine application settings and that exists for most trials, so we did not feel it necessary to draw attention to it in the discussion.

OTHER INFORMATION

23) Registration number and name of trial registry

Does your paper address CONSORT subitem 23? *

Copy and paste relevant sections from the manuscript (include quotes in quotation marks "like this" to indicate direct quotes from your manuscript), or elaborate on this item by providing additional information not in the ms, or briefly explain why the item is not applicable/relevant for your study

n/a this was not a registered trial

24) Where the full trial protocol can be accessed, if available

Does your paper address CONSORT subitem 24? *

Cite a Multimedia Appendix, other reference, or copy and paste relevant sections from the manuscript (include quotes in quotation marks "like this" to indicate direct quotes from your manuscript), or elaborate on this item by providing additional information not in the ms, or briefly explain why the item is not applicable/relevant for your study

contact the authors.

25) Sources of funding and other support (such as supply of drugs), role of funders

Does your paper address CONSORT subitem 25? *

Copy and paste relevant sections from the manuscript (include quotes in quotation marks "like this" to indicate direct quotes from your manuscript), or elaborate on this item by providing additional information not in the ms, or briefly explain why the item is not applicable/relevant for your study

this trial was entirely funded by Hopelab and this is indicated in the CIO section.

X27) Conflicts of Interest (not a CONSORT item)

X27-i) State the relation of the study team towards the system being evaluated

In addition to the usual declaration of interests (financial or otherwise), also state the relation of the study team towards the system being evaluated, i.e., state if the authors/evaluators are distinct from or identical with the developers/sponsors of the intervention.

	1	2	3	4	5	
subitem not at all important	○	○	○	○	◉	essential

Does your paper address subitem X27-i?

Copy and paste relevant sections from the manuscript (include quotes in quotation marks "like this" to indicate direct quotes from your manuscript), or elaborate on this item by providing additional information not in the ms, or briefly explain why the item is not applicable/relevant for your study

detailed in the COI section.

About the CONSORT EHEALTH checklist

As a result of using this checklist, did you make changes in your manuscript? *

- ○ yes, major changes
- ○ yes, minor changes
- ◉ no

What were the most important changes you made as a result of using this checklist?

n/a

How much time did you spend on going through the checklist INCLUDING making changes in your manuscript *

2 hours

As a result of using this checklist, do you think your manuscript has improved? *

- ○ yes
- ◉ no
- ○ Other:

Would you like to become involved in the CONSORT EHEALTH group?

This would involve for example becoming involved in participating in a workshop and writing an "Explanation and Elaboration" document

- ○ yes
- ◉ no
- ○ Other:

Any other comments or questions on CONSORT EHEALTH

Your answer

STOP - Save this form as PDF before you click submit

To generate a record that you filled in this form, we recommend to generate a PDF of this page (on a Mac, simply select "print" and then select "print as PDF") before you submit it.

When you submit your (revised) paper to JMIR, please upload the PDF as supplementary file.

Don't worry if some text in the textboxes is cut off, as we still have the complete information in our database. Thank you!

Final step: Click submit !

Click submit so we have your answers in our database!

SUBMIT

Never submit passwords through Google Forms.

This content is neither created nor endorsed by Google. Report Abuse - Terms of Service - Privacy Policy

Google Forms
